# Electrical Transport of Nb‐Doped MoS_2_ Homojunction P–N Diode: Investigating NDR and Avalanche Effect

**DOI:** 10.1002/smll.202509043

**Published:** 2025-12-24

**Authors:** Ehsan Elahi, Umer Ahsan, Muhammad Farooq Khan, Jamal Aziz, Payal Chauhan, Paweł Piotr Michałowski, Yuan Chen, Goki Eda, Martin Loula, Kalyan Jyoti Sarkar, Zdenek Sofer

**Affiliations:** ^1^ Department of Inorganic Chemistry University of Chemistry and Technology Prague Prague Czech Republic; ^2^ Department of Semiconductor Systems Engineering, Seoul Sejong University Seoul Republic of Korea; ^3^ Chair of Smart Sensor Systems University of Wuppertal Wuppertal Germany; ^4^ Lukasiewicz Research Network ‐ Institute of Microelectronics and Photonics Warsaw Poland; ^5^ Department of Physics National University of Singapore Singapore Singapore; ^6^ Department of Chemistry National University of Singapore Singapore Singapore; ^7^ Centre for Advanced 2D Materials National University of Singapore Singapore Singapore; ^8^ Institute of Organic Chemistry and Biochemistry of the ASCR Prague Czech Republic

**Keywords:** avalanche effect, homojunction, MoS_2_, NDR effect, photodetector

## Abstract

2D transition metal dichalcogenides (TMDCs) are promising candidates for next‐generation nanoelectronics and optoelectronics. Yet, controlling layer number, stacking angle, and interfacial quality in van der Waals (vdW) heterostructures remains challenging, often limiting device performance and reproducibility. Homojunctions formed within a single 2D material can circumvent these issues, but their reliable fabrication and systematic exploration of exotic quantum phenomena remain elusive. Here, we report the fabrication and characterization of a thickness‐modulated lateral p–n homojunction from a single flake of Nb‐doped MoS_2_. This configuration suppresses interface traps without external interface engineering, enabling excellent and highly stable device performance. The diodes exhibit strong rectifying behavior with a rectification ratio of ≈10⁴ and a remarkably low ideality factor (*η* = 1.23). Notably, we observe field‐dependent negative differential resistance (NDR) at low temperatures, offering unique prospects for unconventional electronic applications. The devices also achieve high photoresponsivity (1.09 × 10^3^ A W^−1^), external quantum efficiency (2.16 × 10⁵%), and detectivity (7.5 × 10^10^ Jones). Furthermore, electrical breakdown studies reveal avalanche multiplication at relatively low voltages, enabling high‐performance avalanche photodetectors. Overall, our results demonstrate a simple yet robust approach for probing carrier multiplication in 2D homojunction p–n diodes, underlining the broad potential of TMDCs in advanced optoelectronic and quantum device applications.

## Graphical abstract

Niobium (Nb)‐doped molybdenum disulfide (MoS_2_) p‐type–n‐type (p–n) homojunction diodes are engineered using thickness‐controlled homo‐interfaces. Current–voltage (I–V) characteristics reveal gate‐tunable rectification, wavelength‐dependent photoresponse, and low‐bias switching. Under illumination, impact‐ionization triggers avalanche multiplication, shifting breakdown and enabling sensitive photodetection in two‐dimensional semiconductors.

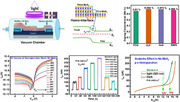

## 1 Introduction

In the age of the Internet of Things (IoT), one of the biggest challenges that scientists are facing is lowering the power consumption of electronic devices [1]. The exceptional electrical and optical characteristics of transition metal dichalcogenides (TMDCs) have garnered significant attention. TMDCs are excellent prospects for field‐effect transistors (FETs), LEDs, and photovoltaics due to their atomic thickness, high carrier mobility, and tunable bandgap [2]. Likewise, the van der Waals (vdW) p–n junctions have been showing a vibrant contribution to real‐world applications of TMDC layered semiconductors [3]. Both the transfer method and direct epitaxial growth have been exploited to establish several vdW's 2D heterostructures [4], including graphene/h‐BN [5], MoS_2_/graphene [6], WS_2_/MoS_2_ [7], GaTe/ReSe_2_ [8], WSe_2_/SnS_2_ [9], and MoS_2_/WSe_2_ heterojunctions [10]. The tunnel FETs (T‐FETs), which are based on carbon nanotube (CNT) [11] and oxide/IV [12] semiconductors, have been thoroughly investigated. 2D) materials present an optimal opportunity for T‐FETs due to their unique properties [13, 14]. The short tunnelling path at the vdW interface enhances the on‐state current, while the absence of dangling bonds at the vdW hetero‐interface helps to minimize charge carrier scattering, thereby improving device performance [15]. While vdW heterostructures (e.g., MoS_2_/graphene, WS_2_/MoS_2_, WSe_2_/SnS_2_) offer strong built‐in fields, their performance is often limited by interface disorder and lattice mismatch [7, 10, 16]. Homojunctions‐engineered by layer‐thickness modulation in TMDC can eliminate interface traps and enable cleaner band‐to‐band tunneling [17, 18], yet reproducible realization of such devices is limited. Unlike other TMDCs, molybdenum disulfide (MoS_2_) possesses a tunable bandgap, making it possible to create multipurpose nanodevices [19]. As the layer number of MoS_2_ drops, its bandgap rises, and it shifts from an indirect (1.3 eV) to a direct bandgap (1.8–1.9 eV) structure. Because of significant mobility, efficient stability and suitable abundance [20, 21], MoS_2_ has been regarded as a potential TMDC candidate for upcoming nano‐electronic devices, including phototransistors [17] and short‐channel transistors [22]. Due to low dark current and better photocurrent, MoS_2_ has recently been sparking as a viable photodetector [23]. It has been recently shown that ferroelectric polarization or an external field can increase photoresponse by further depleting electron carriers and suppressing the dark current. Another tactic to enhance the photocurrent is to create junctions, where the integrated electric field makes it easier to separate the photogenerated electron and hole [17]. The photoresponse was boosted from visible to near‐infrared by the extra interband excitation [24]. The homojunction helps to reduce interlayer scattering which reduces dark current and generates a quick response as compared to heterojunctions where the lattice mismatches will impede photoexcitation and transportation of charges which can minimize the photoresponse [17]. For instance, Lu et al. [18] constructed an MoS_2_ p–n homojunction using nitrogen plasma doping technique and showed that the responsivity increased from 7.5 mA W^−1^ to 48.5 A W^−1^. Though, MoS_2_ typically exhibits n‐type conductivity because of the favored sulfur (S) vacancy. P‐type MoS_2_ can be prepared using a number of techniques, including chemical transport technique [25], ion implantation [26], direct sulfurization [27], and chemical vapor deposition (CVD). For large‐scale growth, CVD has been utilized extensively to examine Nb doping, using NbCl_5_ [28], NaCl mixed Nb_2_O_5_ [17] as the Nb precursors. Nb‐doped MoS_2_ has also recently been grown using the liquid precursor based on niobium oxalate hydrate [29]. Nevertheless, the mobility of the synthesized Nb‐doped MoS_2_ is typically poor. Furthermore, as reported in various study, the effect of Nb doping exhibits significant variations, which is likely attributed to the poor quality of the doped film and challenging doping arrangements, therefore the precise crystal growth of Nb‐doped MoS_2_ is still an issue. Added to this, several initiatives have been undertaken to explore impact ionization in avalanche transistors constructed from 2D materials. Avalanche FETs [30] are becoming significant in electronics owing to their capacity to improve device performance and stability. The avalanche effect is a phenomenon that occurs when there is a significant increase in current resulting from the impact ionization of charge carriers (holes and electrons) within the material. This happens when an electric field (E‐field) over the p–n junction is sufficiently intense, resulting in a fast multiplication of charge carriers. Furthermore, an electrical breakdown is influenced by the electronic band alignment and energy bandgap of 2D materials‐based devices [31]. The electrical breakdown in 2D‐based FETs can be influenced by the thickness of the channel, owing to its quantum confinement, which facilitates a tunable band configuration. Consequently, a detailed study of the mechanisms that drive the electrical breakdown in MoS_2_ under minor electric fields is essential. However, limited information has been revealed related to the avalanche effect of MoS_2_ at strong electric fields, as the atomically thin MoS_2_ channel layer exhibits constrained thermal energy dissipation capacity, resulting in thermal breakdown [32]. Beyond conventional electronics, there is growing interest in harnessing quantum phenomena like impact ionization for avalanche photodetection and negative differential resistance (NDR) for resonant tunneling devices. Avalanche FETs are attractive because impact ionization can dramatically amplify current and optical gain [33, 34]. Yet in 2D semiconductors, avalanche signatures are often masked by thermal breakdown due to inefficient heat dissipation on SiO_2_ and polymer substrates [35]. The dependence of avalanche onset on layer thickness and doping has not been systematically resolved. This knowledge gap limits the design of room‐temperature, power‐efficient avalanche photodetectors. However, power‐efficient and stable avalanche FETs at room temperature are still needed in this era of advanced technology. Similarly, the NDR effect, a signature of resonant tunneling, is highly sensitive to defect states and interfacial quality, making its observation and control in 2D systems a non‐trivial challenge. Thus, the controlled large‐scale crystal growth of Nb‐doped MoS_2_ has remained a problem. Therefore, the NDR effect, photoexcitation characteristics and avalanche effect of the Nb:MoS_2_ homojunction are still unclear.

Herein, we report the synthesis of high‐quality Nb‐doped MoS_2_ bulk crystals via chemical vapor transport (CVT) and the fabrication of lateral homojunctions. The tunability of photoresponse and detectivity in response to the applied voltage, wavelength, and light power has been comprehensively examined in the Nb‐doped MoS_2_ homojunction‐based device. Our findings indicate that, at low temperatures a negative differential resistance (NDR) persists. This indicates an external field may effectively achieve the TFET process under type‐III band configuration, suggesting that gate‐tunable band‐to‐band tunnelling (BTBT) at low temperature is the mechanism due to the optimum interface. We also investigated the electrical breakdown phenomena in Nb‐doped MoS_2_ devices which arise from avalanche multiplication. This comprehensive work provides direction to suppress interfacial traps and to achieve low‐field avalanche breakdown in TMDCs, establishing a pathway for scalable tunneling FETs and high‐sensitivity 2D avalanche photodetectors.

## 2 Experimental Method

### 2.1 Synthesis and Device Fabrication

The bulk crystals of Nb‐doped MoS_2_ were grown via the chemical vapor transport (CVT) process in a quartz glass ampoule via MoCl_5_ as a transport agent. For the synthesis were used molybdenum powder (99.999%, −100 mesh, Quality Key Materials, Co. China) sulfur granules (1–6 mm, 99.9999%, Wuhan Tuocai Technology Co., China) and niobium (−100 mesh, 99.9%, Beijing Metallurgy and Materials Technology Co., China) together with molybdenum pentachloride (99.9%, Strem, USA) as a source of chlorine acting as a transport agent. In the quartz ampoule (50 × 250mm) were placed 50 g of precursors corresponding to MO_0.997_Nb_0.03_S_2_ composition together with 1.0 g of MoCl_5_. Sulfur was used in 2at% excess towards stoichiometry. The ampoule was melt sealed with an oxygen‐hydrogen welding torch under high vacuum using oil diffusion pump with liquid nitrogen trap (base pressure under 1 × 10^−3^ Pa). The ampoule was first heated in a muffle furnace at 500°C for 25 h, at 600°C for 50 h and at 800°C for 50 h. The heating and cooling rate was 1°C min^−1^ and between each temperature step, the mixture was homogenized by shaking for up to 5 min. The generated polycrystalline disulfide was kept in a two‐zone horizontal crystal growth furnace for CVT growth. First, the growth zone was heated at 1000°C and the source zone was placed at 700°C. After the passing of 50 h, the thermal gradient was inverted, the source zone was placed at 1000°C and the growth zone changed to 900°C over a time of 10 days. At the final stage, the growth zone temperature was kept at 500°C for 2 h to eliminate the transport medium and excess sulfur from the growth zone. Above 50% of starting material was transported over the time of 10 days. The ampules were open in the glove box (argon‐filled) and the crystals were placed in the inert atmosphere. The thick‐thin layers of Nb‐Doped MoS_2_ were attained by fundamental mechanical exfoliation techniques, which is a simple scotch tape method. The thick‐thin homojunction was transferred on Si/SiO_2_ by a simple dry transfer method with the help of PDMS. We have used Si/SiO_2_ as substrate with silver (Ag) pre‐patterned as the metal contacts. After transferring the Nb‐doped MoS_2_ on the Si/SiO_2_/Ag substrates, the devices were annealed for two hours at 200°C under high vacuum to make better contact between metal and Nb‐doped MoS_2_ flakes. Natural MoS_2_ crystals were used as an undoped n‐type conductive samples (origin form Krupka, Krusne Hory, Czechia).

## 3 Measurements

### 3.1 SIMS Characterization

Secondary ion mass spectrometry (SIMS) test was performed by means of a CAMECA IMS SC Ultra apparatus with a cesium ion source and positive detector polarity. All signals were recorded as CsX⁺ ions, which are considered semi‐quantitative due to suppression of the matrix effects [36]. Measurements were conducted on five crystals, with elemental composition analyzed at depths of 0.1, 1, 10, and 100 µm as shown in Tables S1–S5. The 1–100 µm range is considered representative of the bulk composition, while the 0.1 µm measurement reflects the near‐surface region.

### 3.2 ICP‐OES Measurements

Bulk 2D crystals of Nb: MoS_2_ were dissolved in 5:1 mixture of HNO3 and HF (both Analpure grade, Analytika s r.o., Czech Republic) in a microwave digestion unit (Magnum II, Ertec, Poland). The solution was then analyzed using an inductively coupled plasma optical emission spectrometer (ICP‐OES) with a radial view of the plasma (Arcos MV, Spectro). Due to complicated spectral background of the molybdenum‐rich matrix, standard addition method was used for measurement and all solutions were spiked with yttrium (Analytika s r.o., Czech Republic) serving as an internal standard. Multiple wavelengths were observed for each element to identify possible spectral interferences. The content of Nb in Nb‐doped MoS_2_ was determined as 0.583%.

### 3.3 STEM Characterization

The exfoliated multilayer Nb‐doped MoS_2_ was transferred onto holey SiN TEM substrate with holes size of 1 µm (TED PAELLA). An aberration‐corrected STEM (JEOL‐ARM200F) furnished with a cold field emission gun and a DCOR probe corrector operating at 80 kV was utilized for HAADF‐STEM imaging. HAADF and MAADF‐STEM images were taken at semi‐angles ranging between 68 and 280 mrad with a probe convergence of≈30 mrad. The energy dispersive X‐ray spectroscopy (EDS) spectra were collected across a 1 µm wide region using STEM.

To verify the quality, nature of crystals and thickness of flakes, Raman spectroscopy and atomic force microscopy (AFM) were utilized. A Raman spectroscopy investigation was performed with a 532 nm wavelength excitation laser with a 1 µm spot. A WITec confocal Raman microscope (WITec alpha300 R, Ulm, Germany) with a 532 nm laser and a spectrometer with a thermoelectrically cooled CCD image sensor was used to record the Raman spectra. AFM was conducted using NT‐MDT Ntegra Spectra in tapping mode; the measurement was carried out at room temperature using a 100× objective lens and a laser power of less than 1.2 mW. A custom‐built 5000 M spectrofluorometer was used to determine photoluminescence excitation (PLE) spectra. The excitation source was a steady‐state laser‐driven Xenon lamp.

### 3.4 Transport Measurements

Transport measurements were performed using a dual‐channel Keysight B1500A semiconductor parameter analyzer at room temperature under high vacuum (10^−6^ bar), the low temperature was attained by liquid nitrogen. The carrier concentration and mobility of the device were evaluated by the DX‐100 Hall effect measurements system by making four points of contact in the Vander Pauw geometry of the bulk crystal. Measurements were conducted at room temperature by sweeping the ±500 mT magnetic field.

## 4 Results and Discussion

### 4.1 Characterization of Material

**FIGURE 1:**
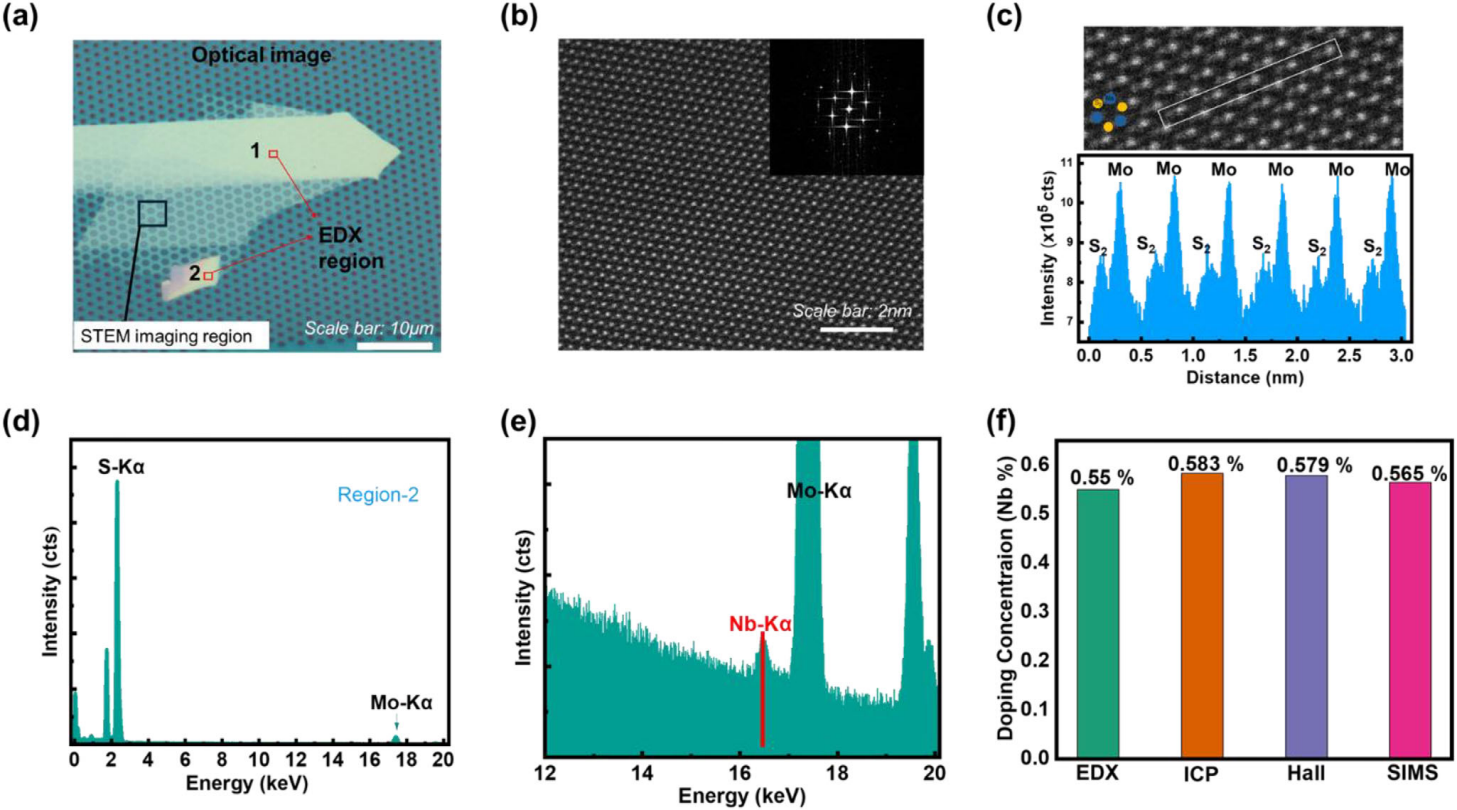
(a) Optical image of Nb‐doped MoS_2_ transferred onto a holey SiN_3_ TEM grid for EDS with region‐1 and region‐2. (b) HAADF STEM image of multilayer Nb‐doped MoS_2_, with the corresponding FFT shown in the inset. (c) Magnified HAADF‐STEM images highlight the integrated image intensity within the white rectangular region, where the Mo column shows consistently higher intensity than the S_2_ column_._ (d) EDS spectrum. (e) EDS spectrum acquired from ≈1 µm^2^ region of the suspended sample (zoom in view). (f) Comparison of EDS, ICP‐OES, Hall measurements and SIMS for Nb concentration.

Figure 1a shows the optical image of Nb‐doped MoS_2_ transferred onto a holey SiN_3_ TEM grid for STEM and EDS with region‐1 and region‐2. Figure 1b shows large‐area HAADF‐STEM images of the multilayer Nb‐doped MoS_2_ sample, revealing the expected hexagonal lattice structure. Due to the similar scattering cross‐sections of Nb and Mo, individual Nb dopants cannot be distinguished from the Mo host atom in Z‐contrast imaging. Nevertheless, we did not identify any Nb substitution at sulfur sites (Figure 1c and Figure S1b,c), which would otherwise result in increased contrast at the S atomic columns. To confirm the incorporation of Nb dopants, we acquired the EDS spectra of the suspended sample (Figure 1d,e). The EDS spectrum exhibits a small peak at ≈16.4 keV, corresponding to the Nb K*α_1_* peak. The low intensity of Nb K*α_1_* is consistent with the nominal Nb doping concentration of 0.6%, which is near the detection limit of our system, suggesting that the Nb concentration in our system is ≈0.55%. The EDS spectrum of region‐1 is shown in Figure S1d,e. SIMS was performed to determine the sample's elemental composition at different depths. Figure S1f presents the near‐surface and bulk compositions, highlighting notable differences, The Nb concentration near the surface is 0.49% and for the bulk crystal is 0.561%. The near‐surface region exhibits a higher O_2_ content, likely due to surface oxidation, and a lower Nb concentration than the bulk. The detailed description of five different samples (crystals) is shown in the Supporting Information in tabular form. It suggests possible Nb diffusion during crystal growth or surface depletion effects. The final device was fabricated using exfoliated flakes, so its composition is expected to be closer to the near‐surface region. The Nb doping percentage examined by EDS, ICP‐OES, Hall and SIMS are shown in Figure 1f, which are nearly identical to each other.

**FIGURE 2:**
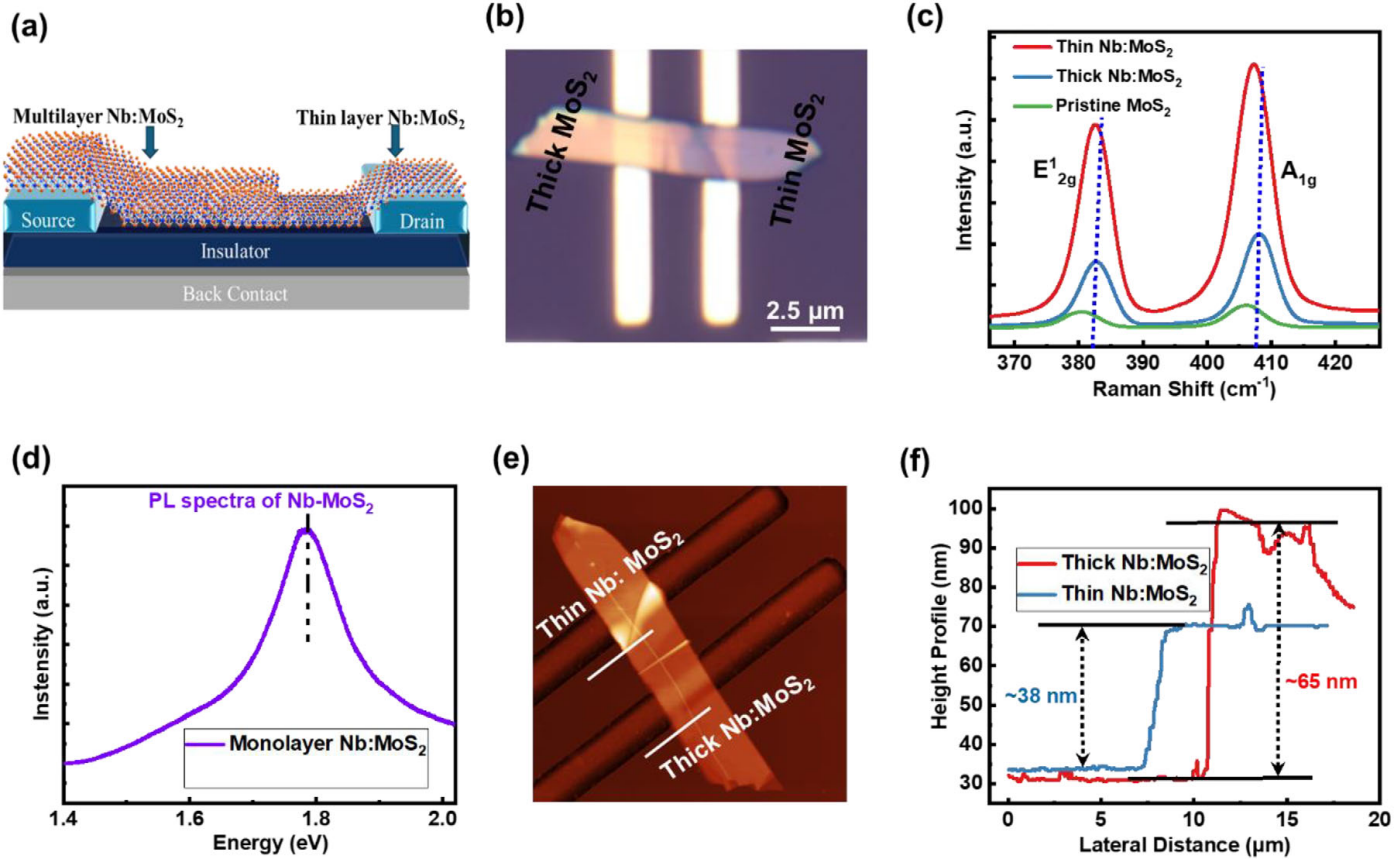
(a) Schematic of the device based on a thick/thin layered Nb‐doped MoS_2_‐based homojunction. (b) Optical image of thick/thin homojunction of Nb‐doped MoS_2_ with silver (Ag) metal electrode. (c) Raman spectra of homojunction Nb‐doped MoS_2_. (d) PL spectra of Nb‐doped MoS_2_ flake showing a strong peak at ≈1.78 eV for the monolayer. (e,f) AFM image of the height profile of thick and thin flake sides.

Figure 2a displays the schematic illustration of the device based on the homojunction of Nb‐doped MoS_2_. Figure 2b shows the optical image of the fabricated device through the conventional viscoelastic stamping method by utilizing PDMS. The desired flakes were transferred on a Si/SiO_2_ substrate that consisted of pre‐patterned metal contacts of silver (Ag). The primary goal was to identify a thick/thin layer homojunction with a well‐defined interface on the substrate. The Raman spectra of pristine MoS_2_, thin and thick sides of Nb‐doped MoS_2_ are represented in Figure 2c, where two dominant modes E^1^_2g_ and A_1g_ are shown respectively [37]. For pristine MoS_2_, E^1^_2g_ is located at 380.4 cm^−1^ and A_1g_ is located at 405.8 cm^−1^, E^1^_2g_ is located at 382.42 cm^−1^ for both Nb‐doped MoS_2_ thin and thick flake sides. The A_1g_ mode of the thin layer is located at 407.12 cm^−1^, but the A_1g_ mode of the thick layered is shifted to a higher wave number which is located at 408.24 cm^−1^. This shows the blue shift and represents the p‐type behavior of the thicker flake side [38]. In Figure 2d, PL spectra reveal a high emission for the mechanically exfoliated monolayer at 1.78 eV (≈ 1.8 eV). The thick flake peak becomes broader which is more obvious, and also shows that Nb‐doping will efficiently tune the electronic structure of MoS_2_ [39]. The reduced PL intensity with greater thickness of Nb‐MoS_2_ represents a transition of band structure [38]. The detailed thickness dependent PL analysis is represented in Figure S1g–i. The blue shift of Raman and PL peaks endorses the tensile strain of the Nb doping effect [40]. This might be attributed to the nonuniform strain field in the crystal domain [41]. Figure 2e,f represents the AFM image and height profile of thin and thick flake sides. X‐ray diffraction (XRD) of bulk pristine MoS_2_ and Nb‐doped MoS_2_ is shown in Figure S2. The XRD pattern of the bulk material shows sharp diffraction peaks, confirming a successful synthesis of MoS_2_ and Nb‐doped MoS_2_ (ICSD 01‐075‐1539). There is a slight shift due to substitutional doping during growth. As Nb has a similar atomic radius to Mo, it is more likely to replace Mo atoms, and as it has slightly higher radii, the structure would have higher interplanar spacing. As a result, there is a slight shift in the XRD patterns.

### 4.2 Electrical Measurement

**FIGURE 3:**
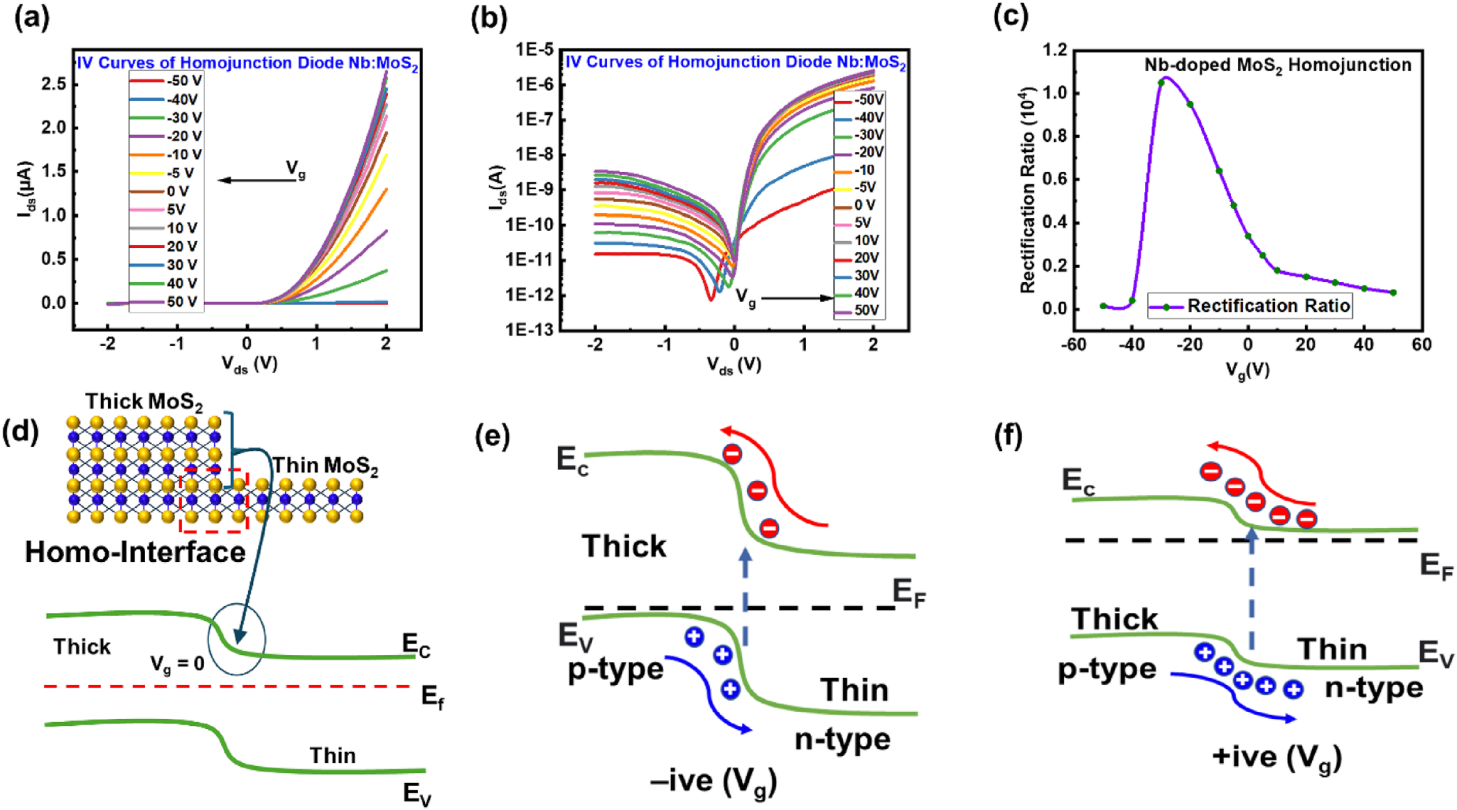
(a) *I*–*V* (linear scale) of the homojunction P–N diode based on Nb‐doped MoS_2_ at various *V*_g_’s. (b) *I*–*V* curves at the logarithmic scale to obtain the rectification ratio (R.R). (c) Variation in the “R.R” with *V*_bg_ applied. (d) Homo‐junction interface based on Nb‐doped MoS_2_. (e,f) Energy band diagram of MoS_2_ homojunction under the varied gate with fixed bias, the transport phenomenon of the carriers of homojunction at different V_g_’s.

We have performed the electrical testing of the device based on the homojunction of Nb‐doped MoS_2_ (thick‐thin). Figure 3a represents the *I*–*V* characteristic curves at different backgated voltages (*V*_g_) with *V*_ds_ ranging from −2 to +2 V. The output characteristics of the vdW homojunction p–n diode are simply examined using the *I*–*V* relation as fundamental p–n diodes [42]

(1) $$I_{ds}=I_{s}\left[\exp\left(\frac{qV}{\eta k_{B\;}T}\right)-1\right]$$

here, *I*_s_ is the saturated current, *q* is the electronic charge, *η* represents the ideality factor, and *k* is the Boltzmann constant. Figure 3b represents the *I*–*V* curves with a logarithmic scale for homojunction p‐n diode. To evaluate the current rectification effect, we checked the rectification ratio (*R*. *R* = *I*_f_/*I*_r_) as the ratio between forward current (*I*_f_) and reverse bias current (*I*_r_). Effectually, sweeping the *V*_g_ yields the highest rectification ratio of 1.05 × 10^4^ at *V*_g_ = −30 V. The alteration in the rectification ratio with *V*_g_ for homojunction is illustrated in Figure 3c. When *V*_g_ < 0 V, the large barrier height is responsible for the reverse bias leakage current. In a similar vein, a negative *V*_g_ broadens the depletion region and raises the barrier height. Additionally, the modulation of the Schottky barrier (SB) height at the metal/TMDC interface by the gate voltage further enhances the rectification ratio. The silver (Ag) was used as metal contact which allows for efficient hole injection into the p‐type Nb‐MoS_2_ thick layer and still has enough electron injection into the thin‐layer n‐type region, Ag can provide reasonably balanced carrier injection due to tunneling effects and reduced contact resistance, facilitating efficient transport across the homojunction. Here, Ag is suitable for both p‐ and n‐type MoS_2_ (homojunction) due to doping, thickness dependency and good interface quality [43, 44]. Meanwhile, in the case of thin n‐type MoS_2_, Ag enables a moderate electron injection Schottky barrier, thus enabling asymmetric charge transport. This barrier inhibits electron conduction in the reverse bias but allows effective hole conduction in the forward bias, thus improving rectification behavior. The inclusion of this asymmetrical charge injection with the band alignment of the homojunction could be the reason for the resulting high rectification ratio (≈10⁴) and ideality factor (1.23), suggesting highly efficient carrier transport. We have checked the stability of the device after a week, and after 2 months, we see minor changes with time as shown in Figure S3a–c. On the other side, device‐2 showed the hole carriers are dominant as characteristics shown in Figure S4a–d, and the threshold voltage changed to a positive region due to Nb doping in MoS_2_, reduced electron concentration and the source–drain current (*I*_ds_) is decreased by two orders [45, 46] and the rectification ratio of device‐2 based on Nb‐doped MoS_2_ reduced to 10^2^. The resistivity, conductivity, carrier concentration, Hall mobility, and Hall coefficient of bulk crystal are represented in Table S6. The ideality factor was extracted in Figure S5a, the lowest value of the ideality factor (*η*) is 1.23, which is attained at *V*_g_ = −30 V. Figure 3d shows thickness modulated homojunction since the energy bandgap (*E*_g_) relies on the number of layers [47], minor *E*_g_ leads to a decrease in the width of the tunneling barrier, and greater *E*_g_ in the channel helps to reduce the off‐state current. Due to homojunction, the problem of interface traps may be ignored without any interface engineering. Irrespective of the homojunction, the edges created on the multilayer side, as illustrated in Figure 3d, should be taken into account since the band configuration at the homojunction is significantly influenced by its edge states. Remarkably, the FET characteristics displayed the shift from p to n‐type behavior when the layer number was reduced [48]. Mechanical exfoliation also introduces strain effects that alter the band structure, shifting the Fermi level towards the conduction band. The p‐type to n‐type transition is significant in an attempt to obtain a naturally occurring homojunction with successful band‐to‐band tunnelling. When the number of layers is decreased, the electron concentration from the S vacancy exceeds the holes from Nb doping, resulting in n‐type behavior. The relationship between the homojunction's thick and thin parts is seen in Figure 3e. As the +ive *V*_g_ increases, the Fermi energy level shifts upward, causing the thin layer to behave eminently n‐type. Figure 3f illustrates how the Fermi energy level keeps shifting higher when *V*_g_ rises to negative. The thick MoS_2_ layer, in this case, shows typical p‐type conduction behavior. Therefore, the band structure becomes steeper as the barrier height between the thick and thin layers rises. The band alignment at *V*g = −30 V is shown in Figure S5b, where the device shows the maximum rectification ratio. The built‐in potential and depletion width are tuned by the gate field, creating a strong, well‐defined p‐n junction with ideal rectification (the gate adds to the built‐in field (*V*_bi_ + |Δ*V*g|), which supports reverse current while preserving efficient forward transport). The rest of the positions give low rectification ratios. At extremely high voltage, current becomes limited by field‐assisted/tunnel paths (and/or contact injection), and the diode asymmetry collapses, yielding low RR a gate‐controlled transition entirely consistent with classical p–n junction electrostatics and with gate‐tunable rectification widely reported in 2D semiconductors [49, 50].

**FIGURE 4:**
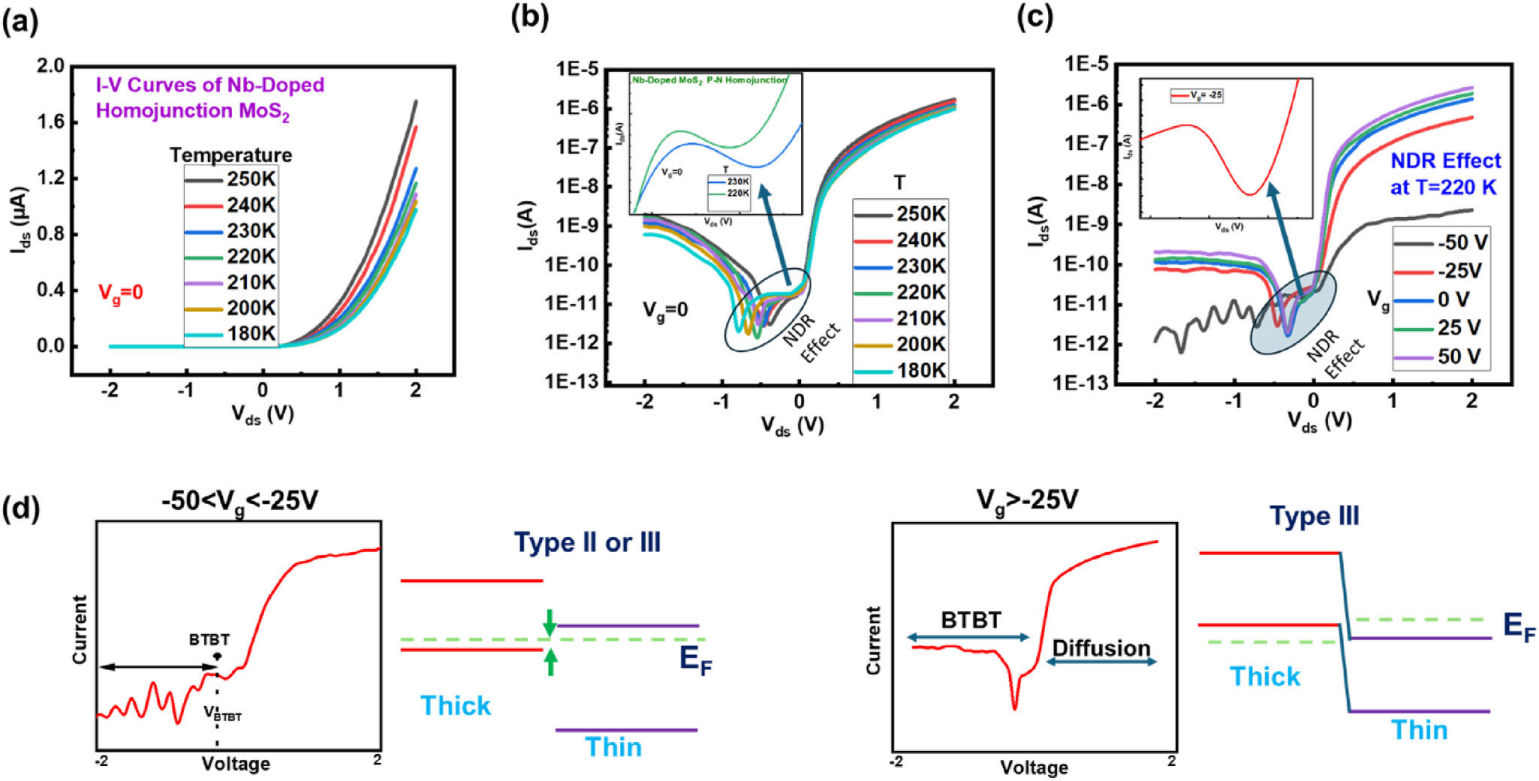
(a) Low‐temperature measurement of the device‐based homojunction Nb‐doped MoS_2_. (b) NDR effect as the function of the temperature: inset shows the zoom‐in view of NDR signal. (c) The NDR effect as the function of *V*_g_: inset represents the zoom‐in view of NDR signal. (d) Energy band diagrams at different *V*_g_’s.

We have examined the *I*–*V* characteristics of the device at low temperatures (180–250 K) with *V*_g_ = 0 and only a minimal decrease in the *I*_ds_ of the device was observed as demonstrated in Figure 4a,b. We have observed the negative differential resistance (NDR) effect in the device as represented in Figure 4c at different *V*_g_ values. The NDR effect arises due to charge trapping in defect states, tunneling mechanisms and quantum confinement effects in the material system [51, 52]. The observed NDR in the homojunction p–n diode of Nb‐doped MoS_2_ originates primarily from the trap‐assisted tunneling (TAT) and thermionic emission mechanisms that dominate carrier transport under specific thermal conditions [53, 54]. At a critical temperature (*T* = 250 K), the thermal energy becomes sufficient to activate charge carriers, leading to a sharp increase in conductivity. The emergence of the NDR effect at 250K can be attributed to an optimal balance between thermal activation and carrier trapping. At 250 K, thermal energy overcomes the potential barrier for charge transport, allowing carriers to escape trapping centers and contributing to conduction which trigger NDR. At this temperature, the shallow and intermediate trap states in the Nb: MoS_2_ bandgap are efficiently activated, facilitating enhanced tunneling and recombination processes. However, as the temperature decreases further (*T* = 160 K), the charge carriers become localized due to reduced thermal energy, suppressing the NDR effect; the detailed form is shown graphically in Figure S6a‐i. Below 160 K, carrier freeze‐out occurs, where thermal energy is insufficient to maintain delocalized transport, leading to carrier localization and suppression of NDR, at cryogenic temperatures, defects and disorder dominate, disrupting ballistic transport needed for NDR [53, 55]. We have also checked the NDR effect at different gate voltage ranges −50 to +50 V and *T* = 220 K. At *V*_g_ = −50 V, the device showed minimal *I*_ds_ and no NDR effect was observed. The NDR effect was prominent at positive gate voltages because of the quantum mechanical tunnelling effect. Typically, NDR is seen at a certain back gate voltage when the energy bands are uniquely aligned (e.g., resonant tunnelling or band‐to‐band tunnelling). This alignment occurs when the potential barrier across the junction and the Fermi levels are aligned by the gate voltage so that the current drops as the voltage rises because there are fewer states available for tunnelling. Nb doping introduces shallow donor states that increase carrier density, modify band alignment by making NDR sensitive to gate voltage and create defect‐assisted tunneling paths which potentially lead to NDR [56, 57]. As schematically shown in Figure 4d, At low temperatures, the device showed no current at *V*_g_ <−50 V, though at −50 V < *V*_g_ < −25 V, a minor BTBT tunneling current seemed at reverse bias, and when *V*_g_ > −25 V an NDR trend appeared by alteration from BTBT current to diffusion current, which is manifest at the forward bias region. These outcomes denote that the band arrangement of the homojunction of Nb‐doped MoS_2_ TFET can be tuned from Type‐II to III by a back gate voltage. Additionally, to clarify the tuning of band configuration, the BTBT onset voltage (*V*_BTBT_) is defined as the voltage at (≈10^−12^ A) value of current and is scaled to the band offset among conduction band maxima (CBM) of thin MoS_2_ and the valence band maximum (VBM) of thick MoS_2_, as represented by the arrow in Figure 4d. As *V*_g_ increased, the band configuration changed from type‐II to type‐III, supporting the idea of the formation of a homojunction in a single flake of Nb‐doped MoS_2_. Furthermore, for the ON‐current, one important thing is that the BTBT current at the reverse bias is less than the diffusion current at the forward bias. Regarding the NDR trend, the forward bias side clearly shows the trace of the changeover point from the BTBT current to the diffusion current. The tunneling current rises, and the Fermi level in the n‐MoS_2_ channel decreases as *V*_g_ increases. As the negative *V*_g_ increased, the NDR trend weakened and eventually vanished beyond −25 V. Hot carrier injection is also a plausible contributing factor for NDR. Especially under high bias hot carriers generated by the high field could be injected into trap states or over barriers, influencing the current. Accumulation of carriers at the junction under bias can also lead to nonlinear current response and may contribute to the observed NDR due to space charge effect. However, TAT is the most consistent explanation, but a combination of mechanisms, potentially including hot carrier effects, may be at play [58].

**FIGURE 5:**
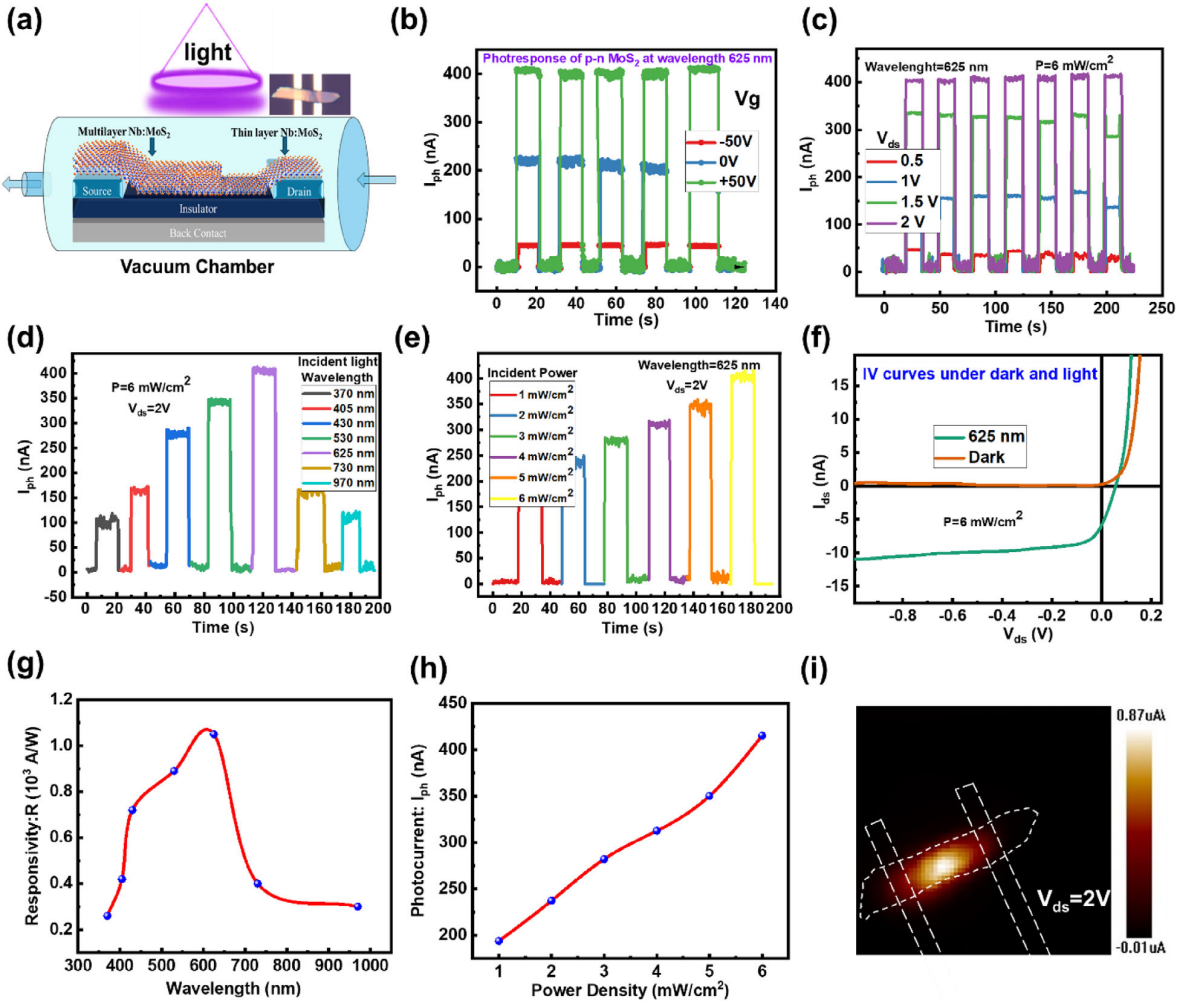
(a) Schematic illustration of the device for photoresponse. (b) Photocurrent at *V*_g_ = −50 V, 0, +50 V with *V*_ds_ = 2 V at wavelength 625 nm with the power 6 mW cm^−2^_._ (c) Photocurrent at *V*_ds_=0.5, 1, 1.5 and 2 V with wavelength 625 nm. (d) Photocurrent observation at various wavelengths (370–970 nm) of incident light. (e) Photocurrent at different powers of the incident light (1–6 mW cm^−2^). (f) *I*–*V* current dark and light to check the *I*_sc_. (g) *R* versus wavelength. (h) *I*_ph_ versus power. (i) Photocurrent mapping by SPCM.

Furthermore, the light‐matter interaction in homojunction Nb‐MoS_2_ devices is extremely intriguing to explore quantum efficiency and photoresponsivity. The optical response of the p‐n diode under the various wavelengths and incident powers is demonstrated. With its tendency for on/off photo‐switching, the photodetector exhibits high stability and reliability. The schematic illustration of the photocurrent investigation setup is shown in Figure 5a, where the FET is positioned in the glass chamber. Light of different wavelengths is directed incident on the homojunction, causing it to generate a large number of electron–hole pairs. These photo‐induced charge carriers tunnel the barrier to produce an efficient photoresponse. As shown in Figure 5b, the maximum photocurrent is about 415 nA at *V*_g_ = +50 V. The photocurrent (*I*_ph_) variation was measured at *V*_ds_ = 2 V and *V*_g_ = −50, 0, and +50 V. Figure 5c shows the photocurrent findings obtained with *V*_ds_ = 0.5 to 2 V. As shown in Figure 5d, we have also examined the photoresponse at several other wavelengths (370, 405, 430, 530, 525, 730, and 970 nm). At 625 nm, the device exhibits a prominent photocurrent and decreases at higher wavelengths. The detailed photoresponse at each wavelength is presented in Figure S7a–f, individually. At 625 nm, the photon energy is around 1.98 eV, which is higher than the bandgap of MoS_2_, allowing for efficient absorption and generation of electron–hole pairs, leading to a significant photocurrent. Occasionally, the photoresponse drops because the semiconductor material has a lower absorption coefficient at longer wavelengths, which leads to less photon absorption and the creation of electron–hole pairs. The decrease in photocurrent at wavelengths higher than 625 nm is primarily due to the reduced photon energy, which becomes insufficient to excite carriers across the bandgap of the Nb‐doped MoS_2_ homojunction. This results in less efficient absorption and lower photocarrier generation. Moreover, doping effects and possible trap states could exacerbate this reduction in photocurrent at longer wavelengths. Additionally, the device's photoresponse was investigated at various incident light power densities (1, 2, 3, 4, 5, and 6 mW cm^−2^). As incident power rises, the device displays higher photocurrent values, as seen in Figure 5e. This implies that at greater power intensities, the rate of electron–hole pair creation exceeds the rate of recombination. Consequently, it suggests that the photogenerated carriers may have a higher percentage of carriers before recombining with defects and capturing sites, which may contribute to the photocurrent. Higher quantum efficiency, which raises the photoresponse by absorbing and converting a larger proportion of input photons into electron–hole pairs, may sometimes result from higher power intensities. The value of short circuit current (*I*_sc_) is 11 nA as shown in Figure 5f. The capability of a device to convert incident light into an electrical signal is measured by its responsivity (*R*). It exemplifies the connection between the input optical power and the resultant electrical output of the photodetector. Responsivity (*R*) of 1.09 × 10^3^ A W^−1^ is calculated by the following equation [59]:

(2) $$R=\frac{I_{\mathrm{ph}}}{PA}$$

where *I*_ph_ is mentioned as photocurrent, *P* is the light power density, and *A* is the effective area of the channel. The EQE value is examined by light absorption and the buildup of photo‐generated carriers. The extracted EQE is about 2.16 × 10^5^ (%) by utilizing the below equation [59]

(3) $$\mathrm{EQE}\;=R\frac{hc}{e\lambda }\;\%$$

*h* is Plank's constant, the speed of light (*c*), an electron's charge (*e*), and *λ* is the wavelength of light respectively. Photogenerated carriers (holes) become trapped at the interface or in defect states for a long time. During this time. They can modulate the channel conductivity, allowing a single photon to cause the circulation of many electrons through the external circuit (photogating). The measured responsivity and EQE thus represent a product of internal quantum efficiency and photoconductive gain. The long carrier lifetime implied by our measured rise/decay times (ms) supports this. Detectivity (D*) is a measure of a photodetector's sensitivity, which may be calculated as D* = *RA* ^1/2^/(2e*I_dark_)^1/2^ [60]. *D* ^*^ is about 7.5x10^10^Jones. The rise/decay times were calculated by fitting the data gained through photocurrent measurements. The rise time is 14 ms and the decay time is 18 ms correspondingly; the detailed findings and equations are signified in Figure S8a,b. The change of *R* w.r.t. wavelength is presented in Figure 5g; the device showed the maximum responsivity at *λ* = 625 nm. The device showed an increasing trend in the photocurrent as the power of incident light increased as shown in Figure 5h. Across the homojunction, there is a locally created built‐in potential caused by the variations in the bandgaps of thick‐thin areas. The separation of charge carriers produced by the incident light is caused by this potential. To assess the source of the produced photocurrent for the fabricated device, scanning photocurrent mapping (SPCM) was employed. *I*_ds_ was measured as a laser beam (*λ* = 625 nm, *P* = 6.73 mW cm^−2^) scanned over the Nb: MoS_2_ homojunction. The photocurrent mapping was attained under the existence of an external bias *V*_ds_ = 2 V, revealed in Figure 5i, in which we can see the effective area of the produced photocurrent. We have added a summary of MoS_2_‐based conventional FETs in Table 1. The exceptionally high EQE and responsivity in our device arise from photoconductive gain mechanisms, such as the photogating effect, where carriers prolong the lifetime of photogenerated holes, enabling multiple electrons to circulate per absorbed photon. This gain is common in 2D photodetectors and is enhanced by the high‐quality, trap‐minimized homojunction interface. While thickness influences gain, the values are consistent with reports on MoS_2_‐based photodetectors under moderate bias (+2 V). The comparison highlights our device's efficient carrier multiplication and low‐voltage operation, rather than thickness alone, as key advantages. In short, superior metrics stem from: (i) gate‐tunable depletion boosting separation, and (ii) internal gain mechanisms verified by dynamics and photo‐assisted avalanche.

**TABLE 1:** Performance evaluation of MoS_2_‐based conventional photodetectors.

Sr.no	Materials	Method	Wavelength (*λ*)	*R* (A W^−1^)	D*(Jones)	Bias voltage (V)	EQE	Refs.
1	Nb‐MoS_2_/MoS_2_	CVD: Nb doped	550 nm	51.4	3 × 10^12^	8	11.65 × 10^3^%	[17]
2	MoS_2_/p‐MoS_2_	Mechanical exfoliation	500 nm	5.07	5 × 10^10^	1.5	7 × 10^3^%	[61]
3	p‐MoS_2_/MoS_2_	CVD/nitrogen doping	532 nm	48.5	≈10^9^	−10	11.301 × 10^3^%	[18]
4	MoS_2_/n‐Si	Precursor solution–annealing	650 nm	11.9	2.1 × 10^10^	−2	—	[24]
5	Intrinsic MoS_2_ phototransistor	CVD	530 nm	0.05	2 × 10^10^	+1	11.7%	[62]
6	Nb: Doped MoS_2_ homojunction	CVT/ Mechanical exfoliation	625 nm	1.09 × 10^3^	7.5 × 10^10^	+2 V	2.16 × 10^5^ %	This work

### 4.3 Thickness‐Dependent Avalanche Effect in Homojunction p–n Diode MoS_2_

**FIGURE 6:**
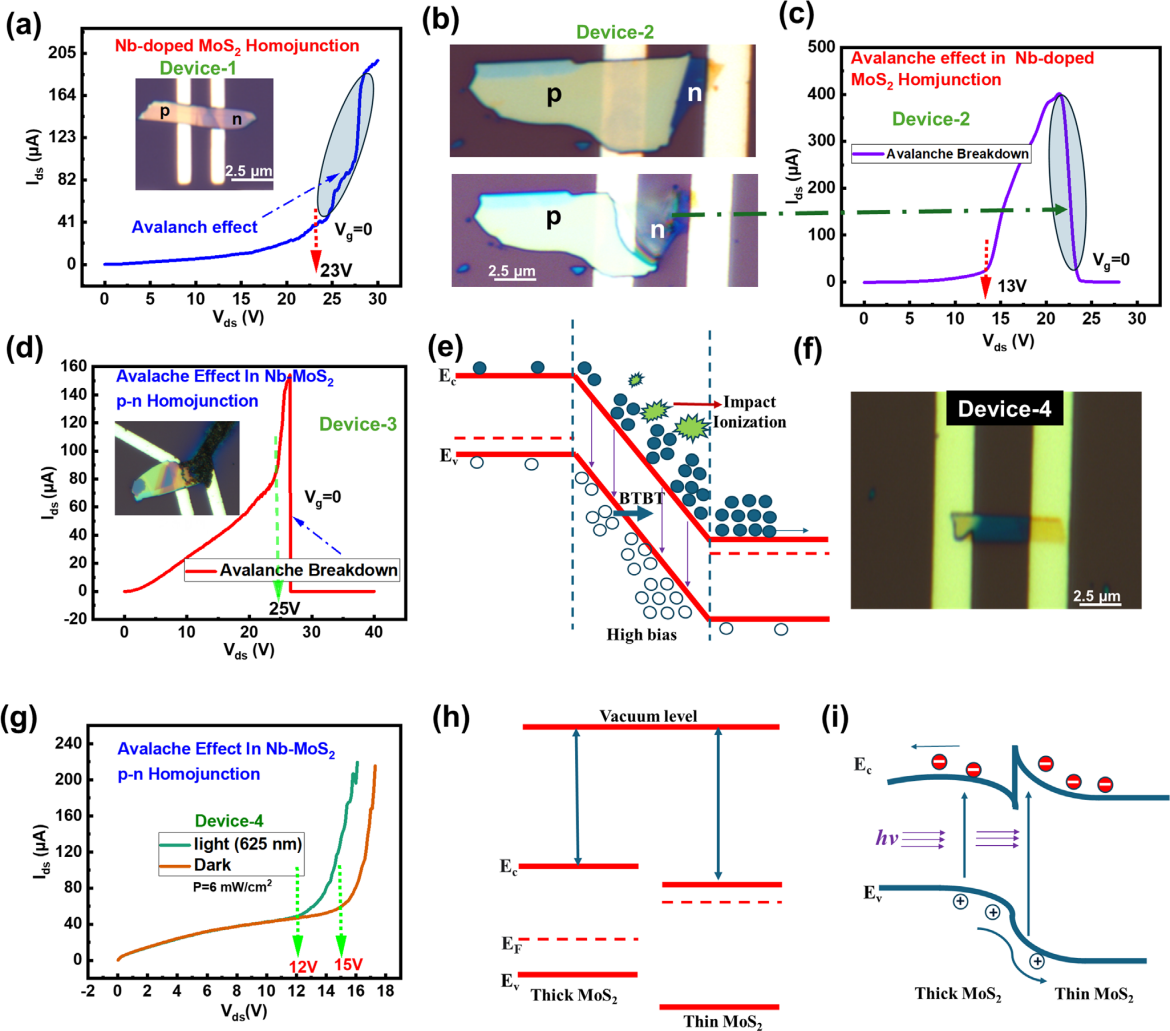
(a) *I*_ds_–*V*_ds_ characteristics of Nb‐doped FET measured at *V*_g_ = 0. The dashed line indicates the breakdown voltage, which is 23 V: inset displays the optical image of device‐1 represents the avalanche effect. (b) Optical image of the device‐2 as fabricated and in burnt state. (c) The avalanche breakdown mechanism of device‐2 is based on the Nb‐doped MoS_2_ channel; the electrical breakdown occurred very early. (d) The avalanche effect in device‐3 shows that the thicker device can sustain the greater voltage, the avalanche phenomenon started at 25 V. (e) Band structure at high bias. (f) The optical image of device‐4, as fabricated. (g) The avalanche mechanism is in‐dark and under radiant light of a wavelength of 625 nm. (h) Band alignment of thick‐thin Nb‐doped MoS_2_ before contact. (i) Energy band diagram and photo‐carrier transport.

Here, we have investigated the electrical breakdown in Nb‐MoS_2_ doped homojunction FETs with various channels and different thicknesses. The examined electrical breakdown of Nb: MoS_2_ FETs under external E‐fields is accredited to the impact ionization in the Nb:MoS_2_‐channel, which is avalanche multiplication. The critical electrical field (*E*_CR_) and impact ionization rate (*α*) are the specific parameters, which are referred as the lowest E‐field needed for the avalanche multiplication and number of electron–hole pairs generated per unit distance travelled by a hot carrier, correspondingly. Specifically, a substantial reliance of ECR and α on the MoS_2_ layer's thickness was directly linked to the quantum confinement effect that was shown in 2D systems. Figure 6a represents the avalanche effect which occurred at 23 V at *V*_g_ = 0, where the avalanche region is highlighted in the circle, inset shows the optical image device that was investigated for avalanche effect. Figure 6b displays the optical images of fabricated device‐2 before and after breakdown. In this device, the avalanche effect was observed at a relatively low voltage. However, as the voltage was further increased, the device experienced a breakdown and subsequently burned out. The fact that breakdown occurs reproducibly at a specific bias and with a steep slope matches an avalanche. We have achieved the impact ionization very early as compared to the bulk devices as shown in Figure 6c. For the thicker device, the electrical breakdown occurred a little bit late at higher voltages (*V*_br_ = 25 V) as shown in Figure 6d; the inset shows the optical image of device‐3. Without any bias, the interdiffusion of the electron and hole emerges at the interface of the homojunction which leads to the bending of the energy band and creates the built‐in electric field (E‐field). After applying high biasing, the potential difference further rises and the built‐in electric field overlaps the initial electric field until the width of the barrier, as shown in Figure 6e, then carrier multiplication occurs which leads to the avalanche effect. It is important to note that after threshold voltage, the avalanche effect and BTBT dominate the current transport. Realizing high performance requires a careful examination of the competing dynamics between avalanche and BBT dark current under high bias [63]. The device‐4 is shown in Figure 6f, which contains the thick‐thin junction of Nb‐doped MoS_2_. In this device, we have checked the avalanche mechanism in dark and under light as well. The avalanche breakdown mechanism occurred early under the laser light, which shows the power‐efficient device. Figure 6g represents the dark current (*I*_dark_) and under illumination with the radiant light of a wavelength of 625 nm at *V*_g_ = 0. *I*_ph_ shows electrical breakdown at a voltage lower than the onset voltage of the dark current's breakdown; the output curves were less steep. This can be attributed to the smaller value of an electric field that the charge carrier feels in the channel arising from the earlier onset of breakdown behavior (smaller voltage). Under illumination (at 625 nm), photocarriers seed the multiplication chain, so the avalanche onset shifts to a lower |*V*| (e.g., from ≈16 V (dark) to ≈12 V (light) as annotated). This is the standard signature of avalanche photomultiplication; fewer volts are required to reach the carrier generation runaway because the initial carrier density is higher. Similar photo‐assisted avalanche behavior is widely seen in MoS_2_/WSe_2_ avalanche photodetectors [64]. Furthermore, increased thickness of the flake causes an increase in the *V*_br_, but doping causes a reduction in the breakdown voltage due to high carrier density. We conclude that the thick‐thin junction reached to early avalanche breakdown as compared to the thicker‐thick (multilayer) homojunction (Device‐1 and 3) of Nb‐doped MoS_2_. The greater electric field concentration in the thin region is the main cause of the earlier avalanche breakdown in the thick‐thin junction of Nb‐doped MoS_2_ as opposed to the thick‐thick (multilayer) junction. Early avalanche breakdown results from the high electric field's ability to accelerate carriers to higher energies more rapidly, which raises the possibility of impact ionization and carrier multiplication. The early breakdown in the thick‐thin structure is also influenced by doping, defect states, and thermal effects. The Fermi level of the thick layer shifted downward as the layer thickness rises as shown in Figure 6h. Figure 6i shows the band bending and photocarrier transport behavior of a thick‐thin junction. The thick/thin layer bends downward/upward suggestively. It gives rise to a potential barrier in the conduction band, which denies the electron transport from thick to thin layers of Nb‐doped MoS_2_. For this reason, the thick layer side gives smaller contribution to the photoresponse of the device. These characteristics lead to understanding the rectifying behavior of device. When the device is positively biased the conduction band of the thin layer is raised and the electron barrier will be smaller, which helps the forward conduction behavior. In the breakdown region, the current boosts to a high level under illumination. Under illumination, the photogenerated carrier can accelerate through the high‐field region, which favors the impact ionization phenomenon [65]. Our study suggests a simple and effective technique for improving the performance of 2D‐TMDC‐based devices. Our work sheds light on the electrical breakdown of Nb: MoS_2_ at both high and low fields. It also contributes to a better understanding of atomically thin avalanche FETs and photodetectors, which is an emerging field in 2D optoelectronics research.

## 5 Conclusion

In summary, we have grown Nb‐doped MoS_2_ by the CVT technique, a method for producing heteroatom‐doped TMDC crystals is offered by the presented Nb‐doping. which uses pre‐Nb metal as the precursor and the p‐type doping. The lateral homojunction Nb‐doped MoS_2_ (thick‐thin) was examined to estimate the current rectification ratio (≈ 10^4^) and photodetection mechanism. Our findings showed that negative differential resistance (NDR) is field‐dependent and occurs at low temperatures. The photodetection and tunability of Nb‐doped MoS_2_ homojunction‐based devices were thoroughly examined. Electrical testing demonstrates that the p‐type doping effect induced by Nb doping in device‐2 leads to a reduction in electron concentration. According to studies on the tunability of MoS_2_ homojunction‐based devices, the external electric field can significantly influence the photoresponse and detectivity. The device shows a maximum photoresponsivity of 1.09 × 10^3^ A W^−1^, the external quantum efficiency (EQE) is 2.16 × 10^5^ % and detectivity (*D* ^*^) is 7.5 × 10^10^ Jones. We also checked the avalanche mechanism in various devices based on Nb‐doped MoS_2_. As a result, the breakdown voltage rises with increasing flake thickness, while the breakdown voltage falls with doping because of the high carrier density. Thus, our work introduces the function of 2D materials in avalanche devices by presenting a straightforward and reliable technique to investigate carrier multiplication in homojunction p–n diodes.

## Author Contributions

E.E., K.J.S., and Z.S. have designed the project. E.E., K.J.S., and U.A. have done device fabrication, electrical measurements, and analyzed the data. P.C. has employed XRD; and P.P.M. has performed SIMS analysis. M.F.K. and J.A. have done a formal analysis of the whole experiment. Y.C. performed TEM imaging under the supervision of G.E. M.L. performed ICP‐OES measurements. Z.S.: supervision, project funding, and growth crystals. All authors wrote the final manuscript. All authors read and approved the final manuscript.

## Conflicts of Interest

The authors declare no conflicts of interest.

## Supporting information




**Supporting Information File1**: smll72001‐sup‐0001‐SuppMat.docx

## Data Availability

The data that support the findings of this study are openly available in Zenodo at https://zenodo.org/records/15649350, reference number 15649350.
