# Supplementary material for: Electrical Transport of Nb‐Doped MoS2 Homojunction P–N Diode: Investigating NDR and Avalanche Effect
Source: Small. 2025 Dec 24;22(9):e09043. doi: 10.1002/smll.202509043 (PMC12895215; doi:10.1002/smll.202509043)
Supplement: Supplementary file 1 — Supporting Information File1: smll72001‐sup‐0001‐SuppMat.docx [file SMLL-22-e09043-s001.docx]

**Supporting Information**

**Electrical Transport of Nb-Doped MoS₂ Homojunction P-N Diode: Investigating NDR and Avalanche Effect**

Ehsan Elahi^1#^, Umer Ahsan^1#^, Muhammad Farooq Khan^2^, Jamal Aziz^3^, Payal Chauhan^1^, Paweł Piotr Michałowski^4^, Yuan Chen^5^, Goki Eda^5,6,7^, Martin Loula^8^, Kalyan Jyoti Sarkar^1*^, Zdenek Sofer^1*^

^1^*Department of Inorganic Chemistry, University of Chemistry and Technology Prague Technická 5, Prague 616628, Czech Republic*

^2^*Department of Electrical Engineering, Sejong University, 209 Neungdong-ro, Gwangjin-gu, Seoul, 05006, South Korea*

*^3^*Chair of Smart Sensor Systems, University of Wuppertal, Wuppertal, Germany

*^4^Lukasiewicz Research Network - Institute of Microelectronics and Photonics, Aleja Lotników 32/46, 02-668 Warsaw, Poland*

*^5^Department of Physics, National University of Singapore, 2 Science Drive 3, Singapore 117551, Singapore*

*^6^Department of Chemistry, National University of Singapore, 3 Science Drive 3, Singapore 117543, Singapore*

*^7^Centre for Advanced 2D Materials, National University of Singapore, Singapore 117546, Singapore*

*^8^Institute of Organic Chemistry and Biochemistry of the ASCR, v.v.i., Flemingovo nám. 2, 166 10 Prague, Czech Republic*

*^#^These two authors contributed equally*

Corresponding Authors (^*^): Kalyan Jyoti Sarkar: sarkark@vscht.cz ; Zdenek Sofer: Zdenek.Sofer@vscht.cz

The photoluminescence (PL) spectra of Nb-doped MoS₂ clearly demonstrate the thickness-dependent bandgap evolution. In the **monolayer** case (**Figure S1(g)**), a strong PL peak appears near 700 nm, corresponding to a direct bandgap of ≈1.78 eV. As the material thickness increases to the **multilayer** regime (**Figure S1(h)**, the PL peak shifts to around 900 nm, giving an effective bandgap of ≈1.38 eV, while the reduced emission intensity reflects the crossover toward an indirect bandgap. For the **bulk-like sample** (**Figure S1(i)**), the PL maximum is observed near 1029.2 nm, corresponding to ≈1.21 eV, which matches the reported indirect bandgap values for bulk MoS₂. This monotonic decrease in bandgap with increasing thickness is further influenced by Nb doping, which induces slight band renormalization and broadening of the emission spectra. These trends are in agreement with previous studies on the optical properties of MoS₂ across different thicknesses

**
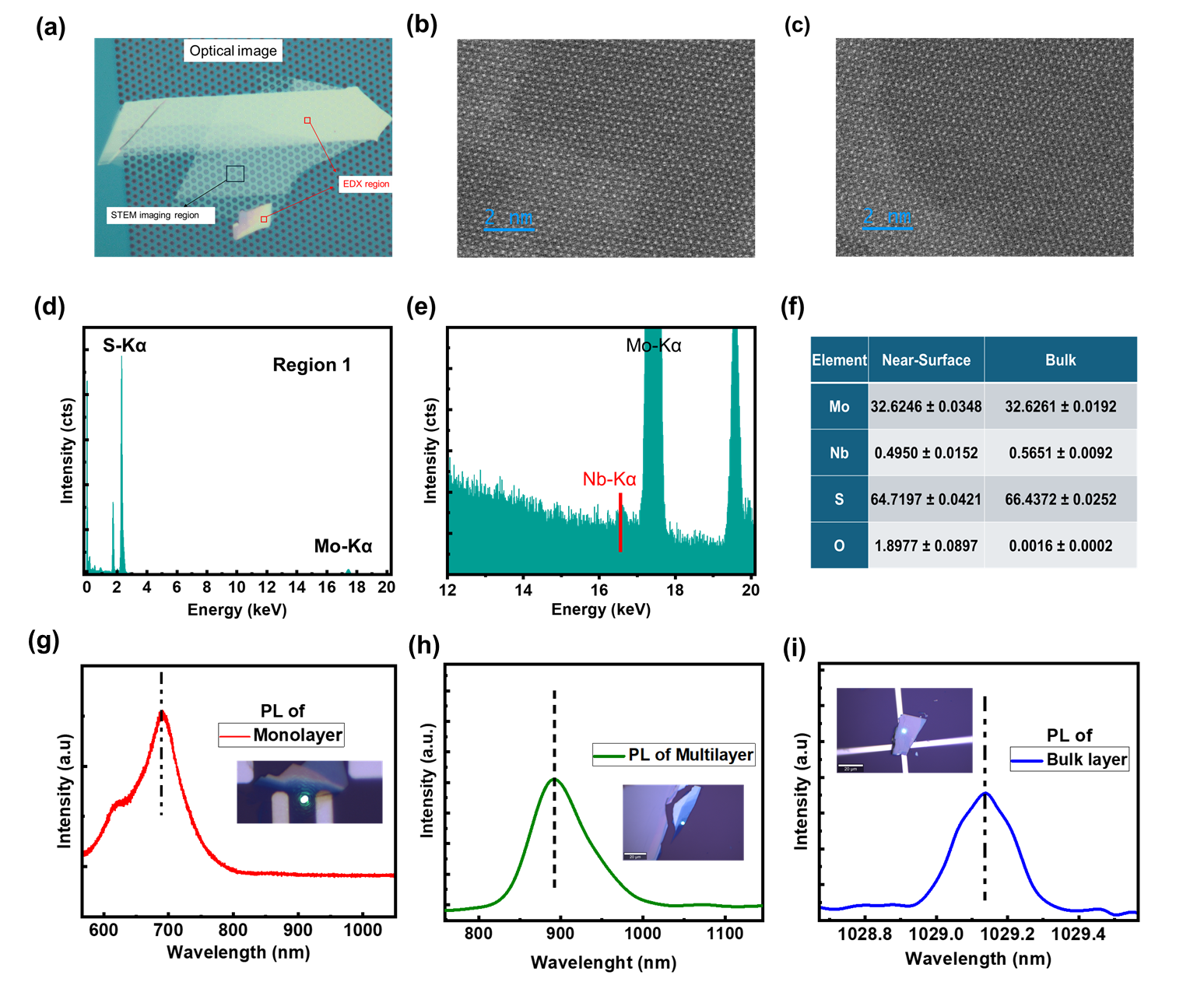
**

**Figure S1:** (a) Optical image of Nb-doped MoS_2_ transferred onto a holey SiN_3_ TEM grid. (b,c) Additional large-area HAADF-STEM images of multilayer Nb-doped MoS_2_ from different regions of the sample. (d) EDX spectrum. (e) EDX spectrum acquired from ~1 µm² region of the suspended sample. (f) Elemental composition of the sample measured by SIMS. (g-i) PL spectra of Nb-doped MoS₂ flakes (monolayer, multilayer, bulk) showing thickness-dependent bandgap red-shift from ~1.78 to ~1.20 eV.

**SIMS results**

The elemental composition of individual samples was measured by SIMS at different depths (0.1 µm, 1 µm, 10 µm, and 100 µm). Each table presents data for a different crystal, highlighting depth-dependent variations.

**Sample 1 (table-S1)**

| **Element** | **0.1 µm** | **1 µm** | **10 µm** | **100 µm** |
| --- | --- | --- | --- | --- |
| Mo | 32.6246 ± 0.0348 | 32.6068 ± 0.0346 | 32.6124 ± 0.0351 | 32.6590 ± 0.0300 |
| Nb | 0.4950 ± 0.0152 | 0.5650 ± 0.0180 | 0.5657 ± 0.0173 | 0.5645 ± 0.0118 |
| S | 64.7197 ± 0.0421 | 66.4900 ± 0.0442 | 66.3883 ± 0.0507 | 66.4333 ± 0.0348 |
| O | 1.8977 ± 0.0897 | 0.0012 ± 0.0004 | 0.0018 ± 0.0004 | 0.0017 ± 0.0004 |

**Sample 2 (table-S2)**

| **Element** | **0.1 µm** | **1 µm** | **10 µm** | **100 µm** |
| --- | --- | --- | --- | --- |
| Mo | 32.6020 ± 0.0770 | 32.7350 ± 0.0670 | 32.5870 ± 0.0550 | 32.7010 ± 0.0550 |
| Nb | 0.4930 ± 0.0220 | 0.5720 ± 0.0370 | 0.5770 ± 0.0460 | 0.5570 ± 0.0170 |
| S | 63.5920 ± 0.0640 | 66.5210 ± 0.0730 | 66.3320 ± 0.0710 | 66.5250 ± 0.0550 |
| O | 2.9620 ± 0.1680 | 0.0011 ± 0.0007 | 0.0016 ± 0.0006 | 0.0025 ± 0.0008 |

**Sample 3 (table-S3)**

| **Element** | **0.1 µm** | **1 µm** | **10 µm** | **100 µm** |
| --- | --- | --- | --- | --- |
| Mo | 32.5550 ± 0.0700 | 32.6920 ± 0.0970 | 32.5470 ± 0.0950 | 32.5570 ± 0.0780 |
| Nb | 0.5060 ± 0.0300 | 0.5470 ± 0.0350 | 0.5540 ± 0.0290 | 0.5540 ± 0.0270 |
| S | 65.3130 ± 0.0630 | 66.4140 ± 0.0660 | 66.3860 ± 0.0940 | 66.4250 ± 0.0670 |
| O | 1.0200 ± 0.1340 | 0.0015 ± 0.0006 | 0.0024 ± 0.0006 | 0.0007 ± 0.0005 |

**Sample 4 (table-S4)**

| **Element** | **0.1 µm** | **1 µm** | **10 µm** | **100 µm** |
| --- | --- | --- | --- | --- |
| Mo | 32.7990 ± 0.0790 | 32.6360 ± 0.0960 | 32.7700 ± 0.0860 | 32.5520 ± 0.0540 |
| Nb | 0.4850 ± 0.0440 | 0.5690 ± 0.0480 | 0.5540 ± 0.0390 | 0.5680 ± 0.0220 |
| S | 65.2540 ± 0.0890 | 66.5350 ± 0.0890 | 66.4470 ± 0.0960 | 66.3500 ± 0.0580 |
| O | 1.2270 ± 0.1200 | 0.0025 ± 0.0006 | 0.0010 ± 0.0008 | 0.0018 ± 0.0006 |

**Sample 5 (table-S5)**

| **Element** | **0.1 µm** | **1 µm** | **10 µm** | **100 µm** |
| --- | --- | --- | --- | --- |
| Mo | 32.5720 ± 0.0990 | 32.4940 ± 0.0580 | 32.5710 ± 0.0730 | 32.7800 ± 0.0600 |
| Nb | 0.5140 ± 0.0310 | 0.5660 ± 0.0480 | 0.5620 ± 0.0160 | 0.5480 ± 0.0330 |
| S | 64.1690 ± 0.0700 | 66.4210 ± 0.0710 | 66.4980 ± 0.0530 | 66.5200 ± 0.0520 |
| O | 2.4410 ± 0.1040 | 0.0019 ± 0.0007 | 0.0012 ± 0.0006 | 0.0024 ± 0.0007 |

**X-Ray Diffraction**

**
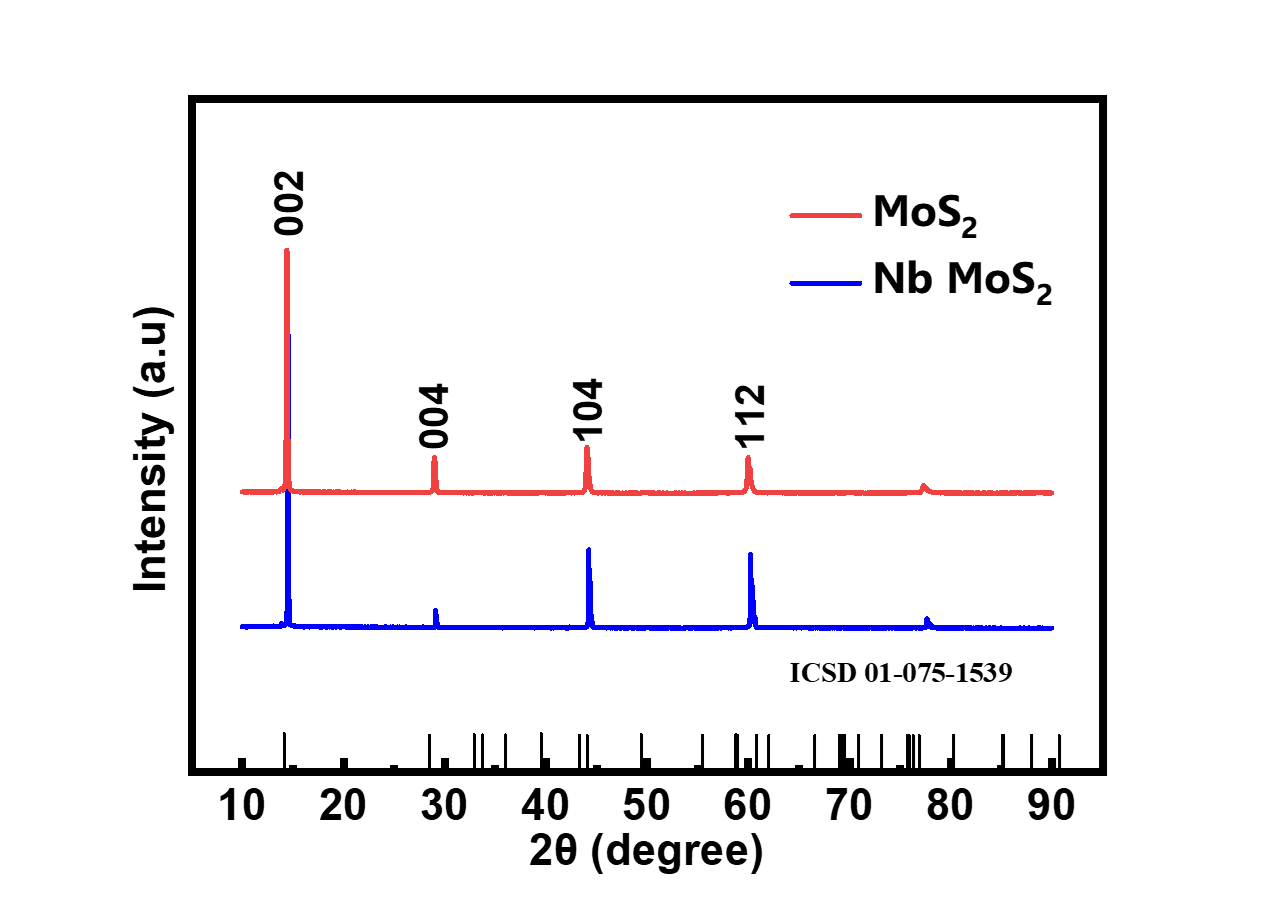
**

**Figure S2:** X-ray diffraction (XRD) of pristine MoS_2_ and Nb-Doped MoS_2_.

**Stability of the device**

**
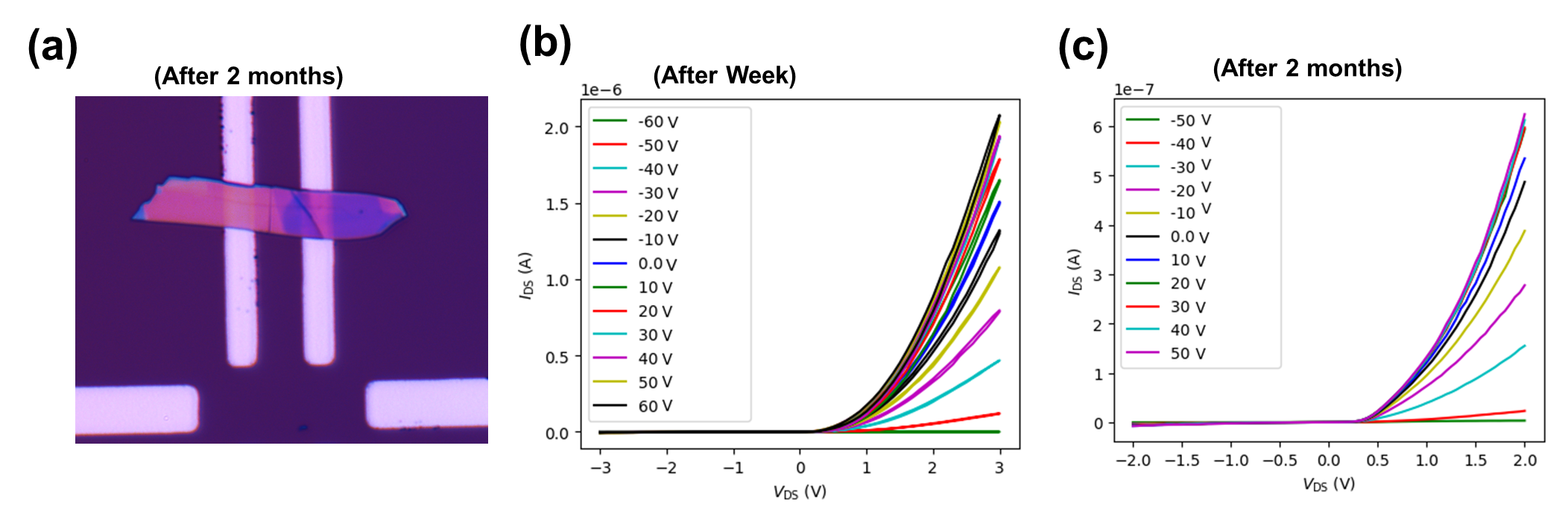
**

**Figure S3:** (a) Optical image of the device after 2 months. (b) I-V curves of the p-n homojunction of Nb-doped MoS_2_ as the function of V_g_, after one week. (c) I-V curves of Nb:MoS_2_ after two months.

**Device-2**

**
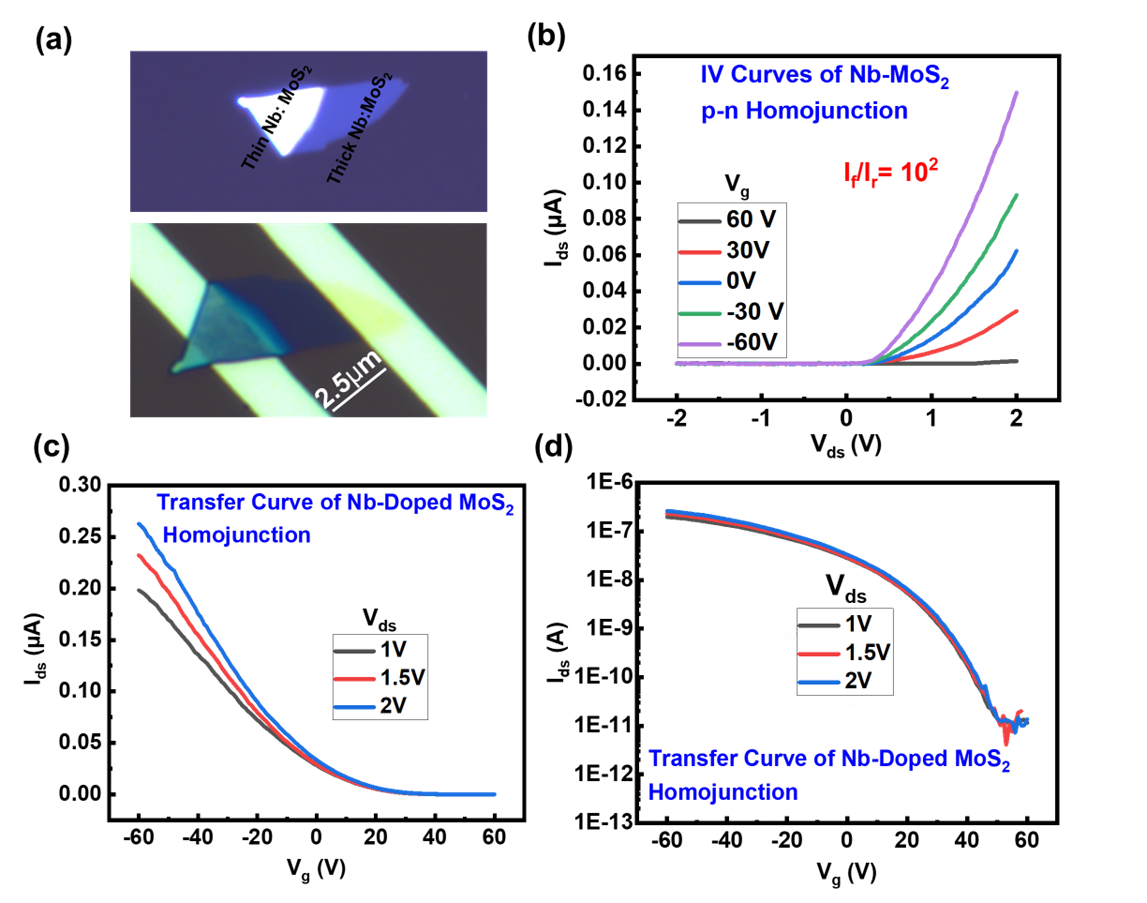
**

**Figure S4:** (a) The optical image of the fabricated device based on the homojunction of Nb-doped MoS_2_. (b) I-V curves of the p-n homojunction of Nb-doped MoS_2_ as the function of V_g_. (c) Transfer curves of Nb: MoS_2_. (d) Transfer curves in logarithmic scale.

**Mobility and carrier concentration of Bulk Nb-doped MoS_2_:**

The carrier concentration value for pristine MoS_2_ is 3.43x10^14^ atoms/cm^3^ and 6.84x10^19^ atoms/cm^3^ for Nb-doped with applied current I=1 mA.

| No | Thickness (cm) | Magnetic  Field (±mT) | Temperature  (K) | Hall Voltage  (mV) | Hall coefficient  （cm³/C） | Bulk carriers concentration  （/cm³） | Sheet carriers concentration  （/cm^2^） | Hall Mobility  (cm^2^/V. s） | Resistivity  （Ω.cm） | Conductivity  （/Ω.cm） |
| --- | --- | --- | --- | --- | --- | --- | --- | --- | --- | --- |
| **1** | 0.117 | 100 | 300 | 2.50E-05 | 0.292527 | 2.14E+19 | 2.50E+18 | 2.840391 | 0.102988 | 9.709851 |
| **2** | 0.117 | 200 | 300 | 3.13E-05 | 0.182821 | 3.42E+19 | 4.00E+18 | 1.777339 | 0.102862 | 9.721739 |
| **3** | 0.117 | 300 | 300 | 0.000106251 | 0.414378 | 1.51E+19 | 1.76E+18 | 4.022516 | 0.103015 | 9.707351 |
| **4** | 0.117 | 400 | 300 | 3.12E-05 | 0.0914 | 6.84E+19 | 8.00E+18 | 6.289136 | 0.102796 | 9.728009 |
| **5** | 0.117 | 500 | 300 | 0.000125006 | 0.292514 | 2.14E+19 | 2.50E+18 | 6.838802 | 0.103041 | 9.704854 |

**Table S6: The characteristics of Nb-doped MoS₂-based bulk crystal**

**Ideality factor calculation**

The ideality factor was calculated for the forward-biased zone by fitting the logarithmic I-V characteristics to the Shockley diode equation [1, 2].

$$I_{D}=I_{S}\left[ \exp\left( \frac{qV}{\eta k_{B}T} \right)-1 \right]$$

Where I_D_ represents the diode current, I_S_ represents the reverse bias saturation current, V denotes the applied voltage, η symbolizes an ideality factor, T signifies temperature, q symbolizes electronic charge, and k_B_ indicates Boltzmann's constant. For applied voltages larger than k_B_T (e.g., > 0.1 V), the term "-1" in the preceding equation can be ignored.

$$\ln\left( I_{D} \right)=\ln\left( I_{S} \right)+ \left( \frac{q}{\eta K_{B}T} \right)V$$

η = $\frac{1}{Slope}\left( \frac{q}{K_{B}T} \right)$

η = $\frac{1}{Slope}\left( \frac{1.6\times{10}^{-19}C}{1.38\times{10}^{-23}JK^{-1} \times300K} \right)$

Slope= 31.34

$\eta=\frac{38.6}{31.34}$ =**1.23** at V_g_= **-30V.**


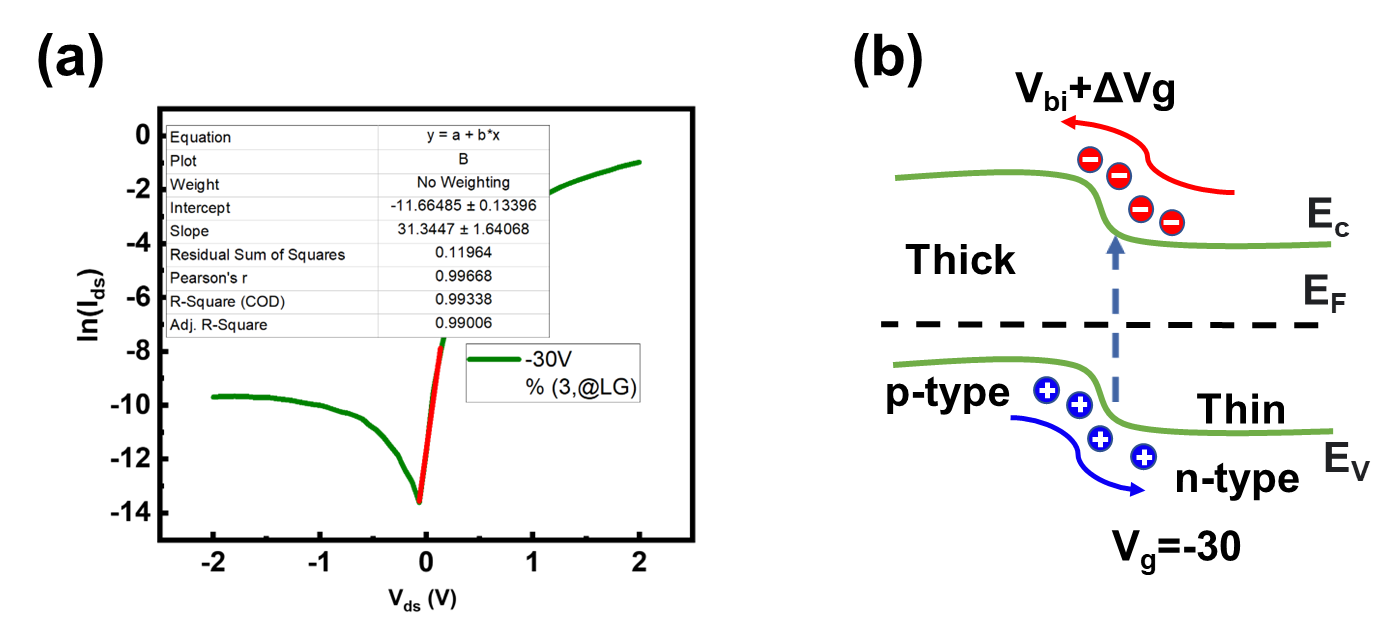


**Figure S5:** Ideality factor calculation at back gate voltage V_g_= -30 V. (b) Gate-controlled band diagrams **Vg**=**−30 V**, illustrate the maximum rectification.

**NDR Effect in Device-1**


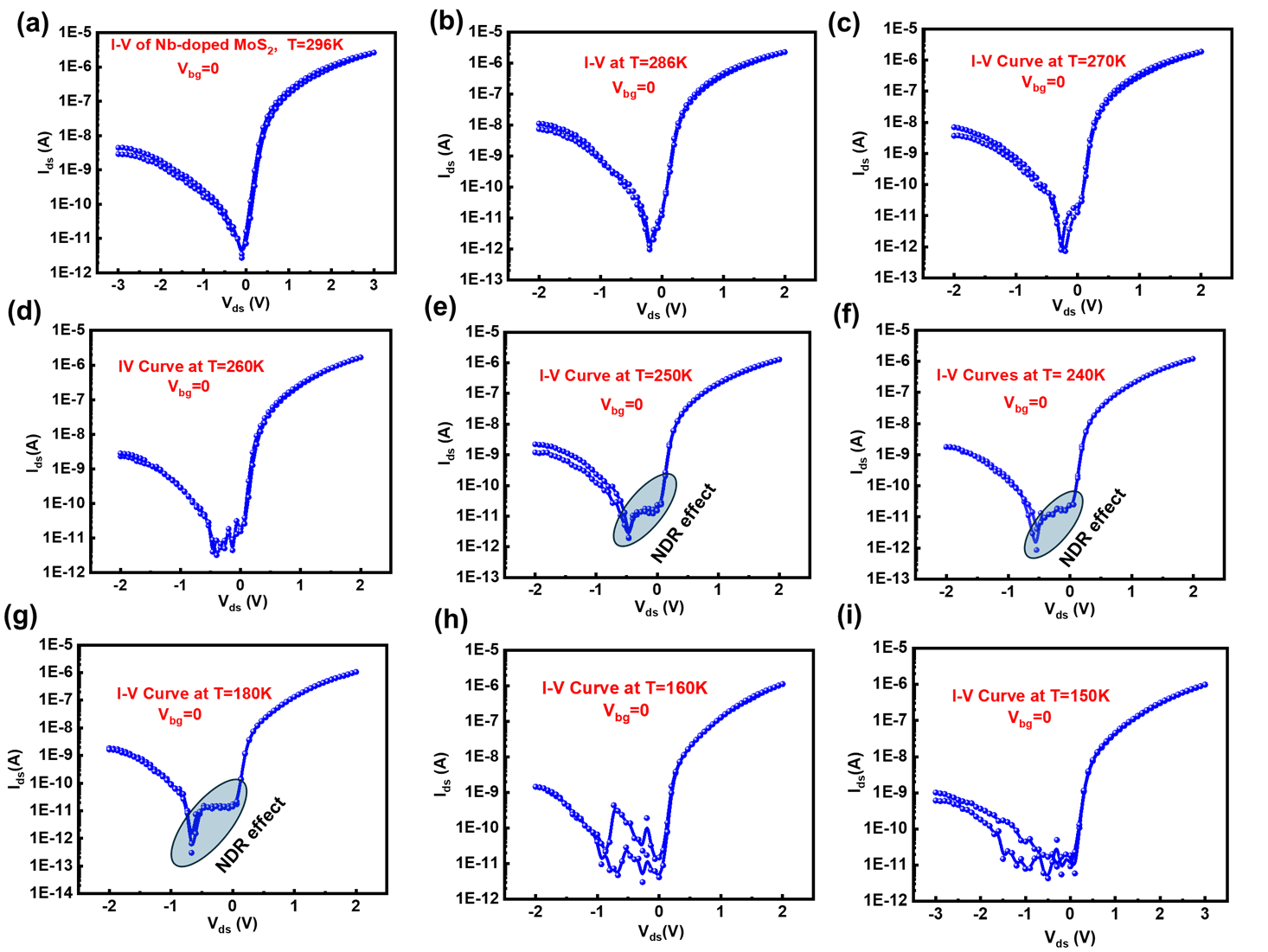


**Figure S6**: (a-i) Investigation of NDR effect in device-1 at different temperatures.

**Photoresponse at different Wavelengths**


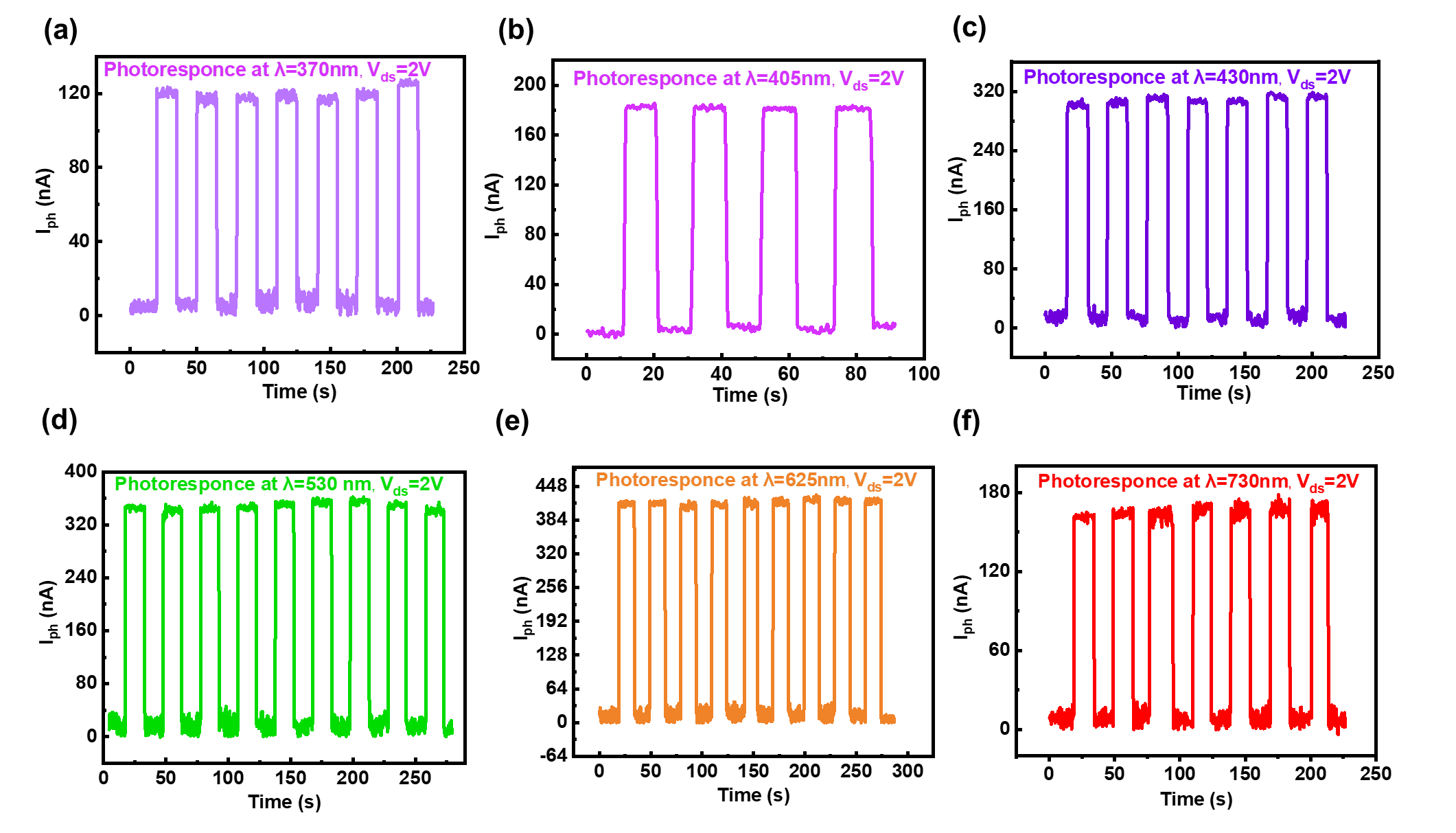


**Figure S7:** (a-f) Photoresponse of the device-1 at different wavelengths (370, 405, 470, 530, 625, 740 nm).

**Rise and decay time**


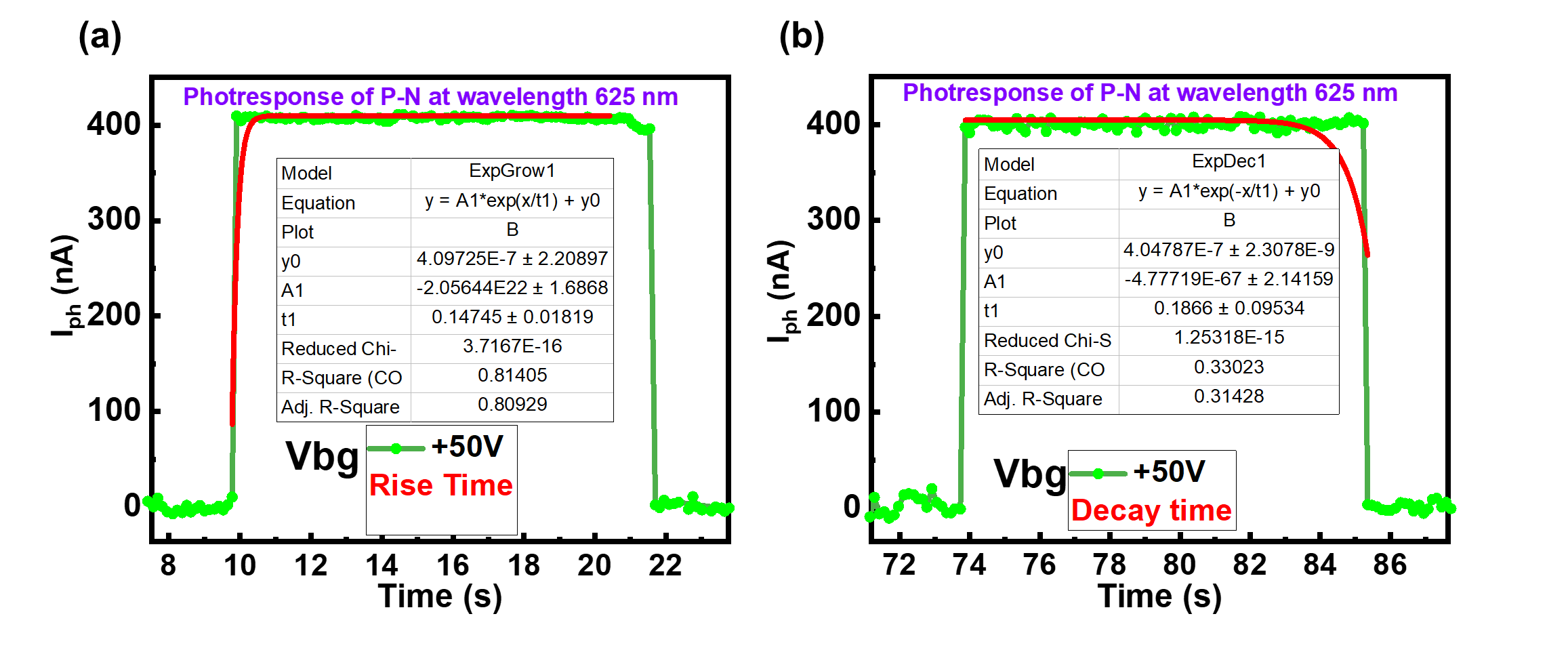


**Figure S8:** (a) Rise time and (b) Decay time of device-1 at wavelength 625 nm.

**References**

1. Martı, A.; Balenzategui, J.; Reyna, R., *Journal of Applied Physics* **1997,** *82* (8), 4067-4075.

2. Elahi, E.; Khan, M. F.; Rehman, S.; Khalil, H. W.; Rehman, M. A.; Kim, D.-k.; Kim, H.; Khan, K.; Shahzad, M.; Iqbal, M. W., *Dalton Transactions* **2020,** *49* (29), 10017-10027.
